# Supplementary material for: Initiating polyketide biosynthesis by on-line methyl esterification
Source: Nat Commun. 2021 Jul 23;12:4499. doi: 10.1038/s41467-021-24846-7 (PMC8302727; doi:10.1038/s41467-021-24846-7)
Supplement: Supplementary file 1 — Supplementary Information [file 41467_2021_24846_MOESM1_ESM.pdf]

Supplementary Information

Initiating Polyketide Biosynthesis by On-line Methyl Esterification

Pengwei Li,[1] Meng Chen,[1,2] Wei Tang,[1,2] Zhengyan Guo,[1] Yuwei Zhang,[1,2] Min Wang,[1,2,3] Geoff P. Horsman,[4] Jin Zhong,[1] Zhaoxin Lu,[5] and Yihua Chen[1,2,*]

[1] State Key Laboratory of Microbial Resources & CAS Key Laboratory of Microbial Physiological and Metabolic Engineering, Institute of Microbiology, Chinese Academy of Sciences, Beijing 100101, China.

[2] University of Chinese Academy of Sciences, Beijing 100049, China.

[3] School of Biotechnology and Health Sciences, Wuyi University, Jiangmen 529020, Guangdong, China.

[4] Department of Chemistry and Biochemistry, Wilfrid Laurier University, Waterloo, ON, N2L3C5, Canada.

[5] College of Food Science and Technology, Nanjing Agriculture University, Nanjing, 210095, China.

*Corresponding author: chenyihua@im.ac.cn

Prof. Yihua Chen

Institute of Microbiology, Chinese Academy of Sciences, Beijing 100101, China

Tel: 86-10-64806121

Supplementary Tables

Supplementary Table 1. Homologous proteins and proposed functions of genes involved in ARTs biosynthesis.

Supplementary Table 2. ^{1}H NMR and ^{13}C NMR data of ART 11B in DMSO-$_{d6}$.

Supplementary Table 3. ^{1}H NMR and ^{13}C NMR data of ART 13B in DMSO-$_{d6}$.

Supplementary Table 4. ^{1}H NMR data of ART 14A in DMSO-$_{d6}$.

Supplementary Table 5. ^{1}H NMR and ^{13}C NMR data of ART D in DMSO-$_{d6}$.

Supplementary Table 6. ^{1}H and ^{13}C NMR data of ART 9B in DMSO-$_{d6}$.

Supplementary Table 7. Incorporation of ^{13}C-labeled acetate into ART 9B as determined by ^{13}C NMR.

Supplementary Table 8. Methyltransferases used for the phylogenetic analysis.

Supplementary Table 9. Bacterial strains and plasmids.

Supplementary Table 10. Primers used in this study.

Supplementary Figures

Supplementary Fig. 1. Spectral data of ART 11B.

Supplementary Fig. 2. Spectral data of ART 13B.

Supplementary Fig. 3. Spectral data of ART 14A.

Supplementary Fig. 4. Tandem MS analysis of ART A and ART 14A.

Supplementary Fig. 5. Sequence alignment of the ACP domains of different ART PKS modules.

Supplementary Fig. 6. Phylogenetic analysis of acyltransferases from different *trans*-AT PKSs.

Supplementary Fig. 7. Spectral data of ART D.

Supplementary Fig. 8. Spectral data of ART 9B.

Supplementary Fig. 9. SDS-PAGE analysis of purified proteins.

Supplementary Fig. 10. The ^{13}C NMR spectra of ART 9B labeled with diverse ^{13}C labeled precursors.

Supplementary Fig. 11. The ^{13}C NMR spectra of ART B labeled with [1-^{13}C] sodium acetate.

Supplementary Fig. 12. HRMS analysis of the production of ART B in *B. subtilis* Δ*art28*/*Bc*-*bioC* (**a**) and in the Art9 assay (**b**).

Supplementary Fig. 13. LC-MS analysis of the *in vitro* assays of Art28 using malonyl-CoA as a substrate.

Supplementary Fig. 14. HPLC analysis of the malonyl-ACP *O*-MT activity of the five Art28 homologues.

Supplementary Fig. 15. The cell membrane permeability was assessed using propidium iodide after exposure of *Staphylococcus aureus* ATCC 6538 to ART B or ART 9B.

Supplementary Fig. 16. Sequence alignment of the KS domains of different ART PKS modules.

Supplementary Fig. 17. The proposed biosynthetic pathway of ARTs based on the one-polyketide-chain assembly model.

Supplementary Fig. 18. Transmembrane region analysis of Art9.

Supplementary Fig. 19. HPLC analysis of the distribution of ARTs in *B. subtilis* fmb60 culture.

Supplementary Fig. 20. Scheme of a biotin biosynthetic pathway.

Supplementary Fig. 21. Construction of *B. subtilis* Δ*art11*, Δ*art(-1)*, and Δ*art(+1)*.

Supplementary Fig. 22. Construction of *B. subtilis* Δ*art1*, Δ*art28*, Δ*art4*, and Δ*art9*.

Supplementary references

Supplementary Table 1. Homologue proteins and proposed functions of genes involved in ARTs biosynthesis.

Genes	Size(aa)	Proposed functions	Protein homologs* (identity/similarity)
orf(-1)	84	hypothetical protein	(PLV31971.1, 100/100)
art1	185	transcription antiterminator	LoaP (WP_069149214.1, 100/100)
art2	318	acyltransferase	(PWU67377.1, 69/84)
art3	425	MATE family efflux transporter	(WP_142506870.1, 83/92)
art4	222	methyltransferase	UbiE (OUM85499.1, 55/76)
art5	259	4'-phosphopantetheinyl transferase	AcpS (PWU67374.1, 75/84)
art6	770	ACP S-malonyltransferase	PksE (CAB13584.3, 60/76)
art7	82	acyl carrier protein	AcpK (CAE01454.1, 56/77)
art8	222	metal-dependent hydrolase	PksB (WP_003231824.1, 51/72)
art9	267	Pimeloyl-ACP methyl ester carboxylesterase	BioH (1M33_A, 33/44)
art10	220	ACP domain	(SMO95506.1, 80/90)
art11	4380	KS-MT-ACP-KS-KR-ACP-KS-ACP-KS-ACP-ACP	PksL (BBB93439.1, 52/67)
art12	939	KS-DH	(SMO94786.1, 84/91)
art13	4927	ACP-KS-DH-KR-ACP-KS-DH-KR-MT-ACP-KS-DH-KR	PksM (SMO94792.1, 81/88)
art14	5059	ACP-KS-KR-MT-ACP-KS-DH-KR-ACP-KS-KR-ACP-KS	PksN (BBB93456.1, 43/62)
art15	1081	DH-ACP-KS	PksL (TCS93647.1, 50/67)
art16	285	DH	(SDN37848.1, 29/47)
art17	2657	MT-ACP-KS-KR-ACP-KS-ACP	PksL (BBB93439.1, 53/68)
art18	405	β-ketoacyl:ACP synthase	PksF (CAB13585.2, 66/81)
art19	419	3-hydroxy-3-methylglutaryl-CoA synthase	PksG (SFJ92592.1, 78/89)
art20	253	enoyl-CoA hydratase (ECH1)	PksH (SMO31772.1, 90/94)
art21	249	enoyl-CoA hydratase (ECH2)	PksI (SMO31790.1, 93/99)
art22	603	ECH-TE	PksR (BBB93450.1, 51/69)
art23	279	sugar phosphate isomerase/epimerase	(KGE20162.1, 31/50)
art24	400	glycosyltransferase	(SMO31801.1, 76/88)
art25	155	hypothetical protein	(SMO31837.1, 80/92)
art26	478	hypothetical protein	(AAU23527.2, 75/90)
art27	466	multidrug efflux pump	(PWU67359.1, 81/90)
art28	273	methyltransferase	UbiE (SMO31860.1,80/91)
orf(+1)	258	sporulation-control protein	Spo0M (WP_148342858.1, 99/99)

*Given in brackets are GenBank accession numbers and percentage of identity/percentage of similarity.

Supplementary Table 2. ^{1}H NMR and ^{13}C NMR data of ART 11B in DMSO-d_6.

No.	δ_H(ppm, J=Hz)	δ_C(ppm)
1		173.1
2	2.22(d, 6.41), 2H	43.5
3	3.86(m)	64.9
4	1.02(m); 1.32(m), 2H	44.0
5	1.88(m)	27.4
6	1.86(m); 1.98(m), 2H	48.3
7		138.0
8	5.82(d, 11.1)	126.0
9	6.36(m)	128.3
10	6.16(dd, 10.6 and 14.7)	130.5
11	6.03(dd, 10.6 and 14.9)	131.2
12	5.70(dd, 8.4 and 14.9)	135.2
13	2.83(m)	44.7
14		132.5
15	6.36(m)	126.2
16	2.02(m)	42.3
17	2.92(m)	87.3
18	1.49(m)	40.0
19	1.03(m); 1.07(m), 2H	33.8
20	1.07 (m); 1.48(m), 2H	28.7
21	1.63(m)	37.6
22	4.69(d, 5.2)	44.4
23		175.4
24		160.1
25		119.1
26	2.80(m)	38.8
27	3.91(brs)	66.8
28	2.49(m); 2.90(m), 2H	31.7
29		145.1
30		117.7
31		161.4
5a	0.78(d, 5.7), 3H	19.1
7a	1.69(s), 3H	16.4
14a	1.63(s), 3H	22.4
18a	1.04(m), 3H	18.4
26a	0.99(d, 6.8), 3H	18.3
30a	1.89(s), 3H	12.3
1´	4.40(d, 7.8)	104.2
2´	4.07(m)	77.4
3´		205.9
4´	3.83(m)	77.3
5´	3.33(m)	71.1
6´	1.32(d, 5.9), 3H	18.6

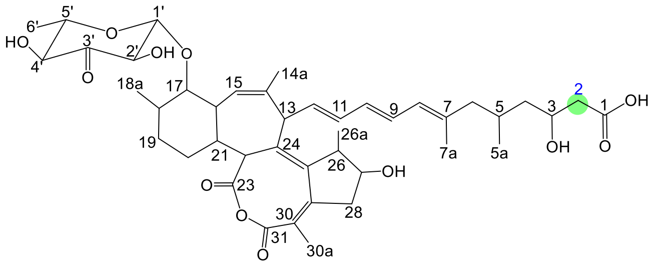

Supplementary Table 3. ^{1}H NMR and ^{13}C NMR data of ART 13B in DMSO-d_6.

No.	δ_H(ppm, J=Hz)	δ_C(ppm)
1		174.3
2	2.20(m)	46.6
3	3.68(m)	69.2
4	0.98(m); 1.34(m), 2H	42.9
5	1.88(m)	27.2
6	1.88(m); 1.99(m), 2H	48.9
7		138.5
8	5.82(d, 11.1)	126.6
9	6.38(dd, 11.1 and 14.7)	128.7
10	6.16(dd, 10.6 and 14.7)	130.5
11	6.05(dd, 10.6 and 14.9)	130.9
12	5.76(dd, 7.4 and 14.9)	136.3
13	3.10(m)	40.1
14	5.63(m)	127.8
15	6.58(m)	131.0
16	2.02(m)	42.5
17	2.95(m)	87.0
18	1.49(m)	40.5
19	0.98(m); 1.09(m), 2H	34.2
20	1.07(m); 1.47(m), 2H	29.1
21	1.63(m)	38.0
22	4.74(d, 5.3)	44.0
23		176.8
24		160.5
25		119.5
26	2.87(m)	38.9
27	3.94(brs)	67.3
28	2.48(m); 2.91(m), 2H	31.7
29		145.4
30		118.3
31		161.8
2a	1.02(m), 3H	13.0
5a	0.77(d,5.7), 3H	19.5
7a	1.70(s), 3H	16.8
18a	1.04(m), 3H	18.9
26a	1.03(m), 3H	18.8
30a	1.89(s), 3H	12.6
1´	4.43(d, 7.9)	104.2
2´	4.07(m)	77.8
3´		206.2
4´	3.83(m)	77.7
5´	3.33(m)	71.7
6´	1.34(d, 5.9), 3H	19.2

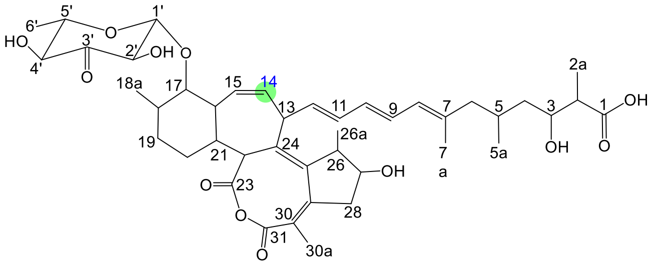

Supplementary Table 4. ^{1}H NMR data of ART 14A in DMSO-d_6.

No.	δ_H(ppm, J=Hz)
1	
2	2.20(m)
3	3.67(m)
4	0.99(m); 1.30(m), 2H
5	1.88(m)
6	1.86(m); 1.98(m), 2H
7	
8	5.82(d, 11.1)
9	6.36(m)
10	6.14(dd, 10.6 and 14.7)
11	6.03(dd, 10.6 and 14.9)
12	5.70(dd, 8.4 and 14.9)
13	2.83(m)
14	
15	6.03(m)
16	1.99(m)
17	3.20(m)
18	1.14(m); 1.18(m), 2H
19	1.48(m); 1.52(m), 2H
20	0.89(m); 1.30(m), 2H
21	1.65(m)
22	4.69(d, 5.2)
23	
24	
25	
26	2.83(m)
27	3.93(brs)
28	2.91(m); 2.94(m), 2H
29	
30	
31	
2a	1.05(m), 3H
5a	0.76(d, 5.7), 3H
7a	1.70(s), 3H
14a	1.65(s), 3H
26a	0.99(d, 6.8), 3H
30a	1.90(s), 3H

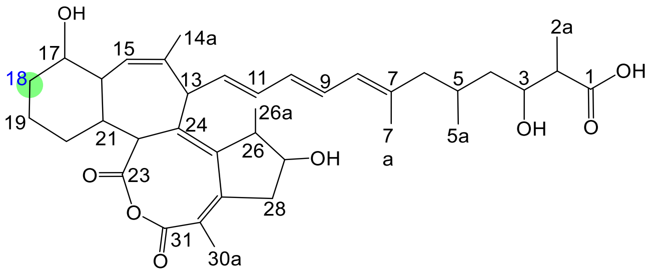

Supplementary Table 5. ^{1}H NMR and ^{13}C NMR data of ART D in DMSO-$_{d6}$.

No.	δ_H(ppm, J=Hz)	δ_C(ppm)
1		176.4
2	2.20(m)	46.3
3	3.68(m)	68.9
4	1.04(m); 1.34(m), 2H	42.2
5	1.88(m)	27.0
6	1.86(m); 1.98(m), 2H	48.4
7		138.2
8	5.82(d, 11.1)	126.3
9	6.38(m)	128.5
10	6.15(dd, 10.6 and 14.7)	131.1
11	6.05(m)	131.5
12	5.68(dd, 8.5 and 14.9)	136.1
13	2.79(m)	44.6
14		132.4
15	6.42(m)	127.7
16	2.08(m)	42.3
17	2.95(m)	87.3
18	1.49(m)	40.4
19	1.03(m); 1.05(m), 2H	33.7
20	1.09(m); 1.49(m), 2H	29.0
21	1.62(m)	37.6
22	4.68(d, 5,2)	44.0
23		181.4
24		159.8
25		118.9
26	2.85(m)	38.8
27	3.86(brs)	67.7
28	2.51(m); 2.90(m), 2H	33.3
29		145.4
30	6.01(m)	110.9
31		160.1
2a	1.03(m), 3H	12.7
5a	0.78(d,5.6), 3H	19.2
7a	1.70(s), 3H	16.4
14a	1.62(s), 3H	22.6
18a	1.04(m), 3H	18.6
26a	0.98(m) 3H	18.3
1´	4.43(d, 7.9)	104.2
2´	4.08(m)	77.4
3´		205.9
4´	3.83(m)	77.3
5´	3.33(m)	72.5
6´	1.32(d, 6.0), 3H	18.8

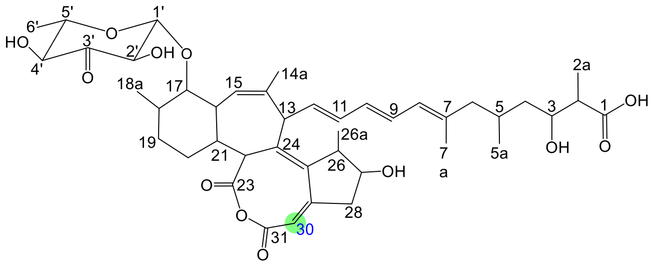

Supplementary Table 6. ^{1}H and ^{13}C NMR data of ART 9B in DMSO-$_{d6}$.

No.	δ_H(ppm, *J*=Hz)	δ_C(ppm)
1		175.4
2	2.34(m) 1H	46.5
3	3.69(s) 1H	69.3
4	1.33(m) 2H	42.7
5	1.87(m) 1H	27.4
6	1.86(m); 1.98(m), 2H	48.8
7		138.4
8	5.81(d, 11.1) 1H	126.7
9	6.38(m) 1H	128.7
10	6.16(dd, 10.6 and 14.7)	130.9
11	6.03(dd, 10.6 and 14.9)	131.6
12	5.70(dd, 8.5 and 14.9)	135.7
13	2.83(m) (1H)	45.1
14		132.9
15	6.40(m) 1H	126.5
16	2.01(m) 1H	42.5
17	2.94(m) (1H)	87.7
18	1.50(m) (1H)	40.0
19	1.03(m); 1.08(m), 2H	33.6
20	1.07(m); 1.50(m), 2H	29.1
21	1.64(m) (1H)	38.0
22	4.69(d, 5.2) 1H	44.8
23		175.8
24		160.5
25		119.5
26	2.82(m) (1H)	39.6
27	3.92(brs) (1H)	67.2
28	2.49(m); 2.90(m), 2H	32.2
29		145.5
30		118.2
31		161.9
1a	3.59(s), 3H	51.7
2a	1.05(m), 3H	12.7
5a	0.78(d, 5,6), 3H	19.2
7a	1.69(s), 3H	16.9
14a	1.64(m), 3H	22.8
18a	1.03(m), 3H	18.9
26a	1.00(d, 6.8), 3H	18.7
30a	1.90(s), 3H	12.7
1´	4.41(d, 7.8)	104.6
2´	4.08(m) 1H	77.8
3´		206.3
4´	3.84(m)	77.7
5´	3.33(m) 1H	71.6
6´	1.33(m), 3H	19.0

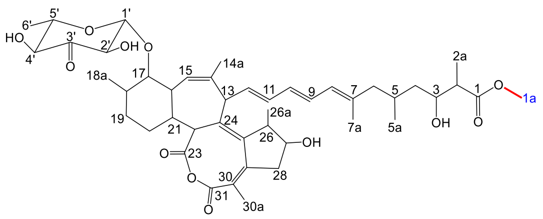

Supplementary Table 7. Incorporation of ^{13}C-labeled acetate into ART 9B as determined by ^{13}C NMR.

No.	^{13}C chemical shift (δ)	Relative intensity[a]	
		[1-^{13}C] acetate	[2-^{13}C] acetate
1	175.4	0.8	0.8
2	46.5	1.0	14.5
3	69.3	3.4	0.7
4	42.7	0.5	8.8
5	27.4	2.1	0.6
6	48.8	0.5	9.8
7	138.4	1.7	0.5
8	126.7	0.5	9.2
9	128.7	2.0	0.6
10	130.9	0.5	8.2
11	131.6	1.6	0.6
12	135.7	0.4	8.2
13	45.1	2.3	0.5
14	132.9	0.5	6.7
15	126.5	1.6	0.5
16	42.5	0.4	11.8
17	87.7	2.3	0.4
18	(40.0)[b]	nd	nd
19	33.6	2.5	0.8
20	29.1	0.5	10.2
21	38.0	1.8	0.7
22	44.8	0.4	9.1
23	175.8	1.5	0.5
24	160.5	2.1	0.5
25	119.5	0.4	6.9
26	(39.6)[b]	nd	nd
27	67.2	2.5	0.7
28	32.2	0.4	10.0
29	145.5	1.8	0.6
30	118.2	0.5	6.9
31	161.9	1.9	0.6
1a	51.7	1.1	1.3
2a	12.7[d]	2.1	2.5
5a	19.2	1.1	14.4
7a	16.9	0.6	10.2
14a	22.8[c]	1.0	1.0
18a	18.9	1.2	1.2
26a	18.7	1.1	1.5
30a	12.7[d]	2.1	2.5
1’	104.6	0.7	0.9
2’	77.8	0.8	0.9
3’	206.3	0.5	0.7
4’	77.7	0.8	0.9
5’	71.6	0.9	1.0
6’	19.0	1.2	1.5

a: the signals enriched by [1-^{13}C] acetate or [2-^{13}C] acetate are indicated with red.

b: overlapped with the solvent peak.

c: signal at δ_c 22.8 was defined as 1.0.

d: chemical shifts of C-2a and C-30a were overlapped at δ_c 12.7.

Supplementary Table 8. Methyltransferases used for the phylogenetic analysis.

MT	Source	Accession number
**malonyl-ACP *O*-MTs**		
Art28	*Bacillus subtilis* fmb60	OEI73477
Thd235	*Thermoactinomyces daqus* H-18	WP_033101217
Bac390	*Bacillus cereus* BAG5X1-1	WP_007932545
Pas675	*Paenibacillus* sp. Mc5Re-14	WP_090739063
Brf335	*Brevibacillus formosus* DSM 9885	WP_047068491
Clp905	*Clostridium papyrosolvens* DSM 2782	WP_004618171
Pef340	*Pelosinus fermentans* DSM 17108	WP_007932545
Soc045	*Sorangium cellulosum* So ce836	AUX33056
Des352	*Desulfoluna spongiiphila* AA1	SCX96925
Tet660	*Teredinibacter turnerae* T7902	WP_018277530
Mis985	*Micromonospora* sp. ATCC 39149	WP_007076119
Aci145	*Actinomyces israelii* DSM 43320	WP_052374539
Dis505	*Dictyobacter* sp. Uno17	WP_149400179
MTtype11	*Gracilibacillus dipsosauri* ATCC 700347	WP_109985211
***C*-MTs**		
Art4	*Bacillus subtilis* fmb60	OEI73453
BtUbiE	*Bacillus thermozeamaize* ATCC BAA-739	OUM85499
DsUbiE	*Desmospora* sp. 8437	EGK11618
PpUbiE	*Pseudomonas putida* BIRD-1	ADR62353
KpUbiE	*Klebsiella pneumoniae* Kp52.145	CDO16246
**BioCs**		
BcBioC	*Bacillus cereus* ATCC 10987	WP_000608937
BsBioC	*Bacillus anthracis* HDZK-BYSB7	QBJ69759
EcBioC	*Escherichia coli* K-12	NP_415298
RhBioC	*Rhizobium* sp. SLBN-4	WP_142012653
PfBioC	*Pseudomonas fluorescens* F113	AEV65368
***O*-MTs**		
CorH	*Corallococcus coralloides* B035	QAT84738
MerK	*Melittangium boletus* DSM 14713	WP_095982058
CtaK	*Cystobacter fuscus* DSM 2262	AAW03333
***N*-MT domains**		
SimAMT1	SimA in cyclosporine biosynthesis	CAA82227
TubBMT	TubB in tubulysin biosynthesis	CAF05647
TxtAMT	thaxtomin synthetase A	AAG27087
***α*-*C*-MT domains**		
CurJ	curacin A biosynthesis	5THZ
LovbMT	LovB in lovastatin biosynthesis	Q9Y8A5
Art11MT	Art11 in aurantinin biosynthesis	OEI73460
Art13MT	Art13 in aurantinin biosynthesis	OEI73462
Art14MT	Art14 in aurantinin biosynthesis	OEI73463
Art17MT	Art17 in aurantinin biosynthesis	OEI73466

Supplementary Table 9. Bacterial strains and plasmids.

**Strains or plasmids**	**Characteristics***	**Reference or source**
Escherichia coli		
DH5α	General cloning host	Invitrogen
BL21 (DE3)	Host for protein expression	Novagen
Bacillus		
B. subtilis fmb60	Aurantinins producing wild-type strain	1
**Plasmids**		
pRN5101	Erm^r, Amp^r, used for inactivation of genes or MT domains	2
pHY300P$_{aprN}$	Tet^r, Amp^r, used for expressing genes in *B. subtilis* fmb60	2
pHY300P$_{aprN}$::*art28*	Tet^r, Amp^r, used for complementing gene *art28* to mutant Δ*art28*	This work
pHY300P$_{aprN}$::*Bc-bioC*	Tet^r, Amp^r, used for expressing gene *Bc-bioC* in mutant Δ*art28*	This work
pET28a	Kan^r, protein production vector	Novagen
pET28a::*art9*	Kan^r, for producing *N*-His$_6$-tagged Art9	This work
pET28a::*art28*	Kan^r, for producing *N*-His$_6$-tagged Art28	This work
pET28a::*art10acp*	Kan^r, for producing *N*-His$_6$-tagged ACP$_{Art10}$	This work
pTGE33	Kan^r, for producing *N*-His$_6$-tagged Sfp	3
pET28a::*clp905*	Kan^r, for producing *N*-His$_6$-tagged Clp905	This work
pET28a::*pas675*	Kan^r, for producing *N*-His$_6$-tagged Pas675	This work
pET28a::*brf335*	Kan^r, for producing *N*-His$_6$-tagged Brf335	This work
pET28a::*thd235*	Kan^r, for producing *N*-His$_6$-tagged Thd235	This work
pET28a::*bac390*	Kan^r, for producing *N*-His$_6$-tagged Bac390	This work

*Erm^r, erythromycin resistance; Amp^r, ampicillin resistance; Tet^r, tetracycline resistance; Kan^r, kanamycin resistance.

Supplementary Table 10. Primers used in this study.

Primers	Sequences (5′ to 3′)*	Uses
11-L-F<br>11-L-F	aaagacataatcgatAAGCTTggcgagacaccagaagaact<br>aatcgccttcttctgtgtcatcggcaaccaaagaggatgaac	Amplification of the upstream region of *art11* (*Hin*dIII)
tetR-F<br>tetR-R	gttcatcctctttggttgccgatgacacagaagaaggcgatt<br>gtcatataagttggttgcctcgtcccaaagttgatcccttaacg	Amplification of the tetracycline resistance gene *tetR*
11-R-F<br>11-R-R	cgttaagggatcaactttgggacgaggcaaccaacttatatgac<br>gaagagcttttataaAAGCTTtacttcgctctaaggctatctg	Amplification of the downstream region of *art11* (*Hin*dIII)
11ver-F<br>Tver-R<br>Tver-F<br>11ver-R	cggctactgttgatgcatcct<br>ccgttcccaattccacatt<br>tatcagagggaacaggtattg<br>tgggtacccgtgccatgcat	Confirmation of genotype of *B. subtilis* Δ*art11*
1-L-F<br>1-L-R	aaagacataatcgatAAGCTTtaaggtcggtgttactgtgg<br>caactcccacatcaaccaacacaatttcttcttgacctg	Amplification of the upstream region of *art1* (*Hin*dIII)
1-R-F<br>1-R-R	caggtcaagaagaaattgtgttggttgatgtgggagttg<br>taaactaccgcattaAAGCTTaatgagctttggctaggttg	Amplification of the downstream region of *art1* (*Hin*dIII)
4-L-F<br>4-L-R	aaagacataatcgatAAGCTTtattgcgattgccacagct<br>acatgtctcaaagttactctcgttcattcccggtcccttg	Amplification of the upstream region of *art4*(*Hin*dIII)
4-R-F<br>4-R-R	caagggaccgggaatgaacgagagtaactttgagacatgt<br>taaactaccgcattaAAGCTTtataacatcgggttgttggc	Amplification of the downstream region of *art4* (*Hin*dIII)
9-L-F<br>9-L-R	aaagacataatcgatAAGCTTttaggtgccaattccgtagatc<br>cccatcagtgcccaataccaaaacaaagagaagtggtggtgttt	Amplification of the upstream region of *art9* (*Hin*dIII)
9-R-F<br>9-R-R	aaacaccaccacttctctttgttttggtattgggcactgatggg<br>taaactaccgcattaAAGCTTcagcagggtaatcggcatcc	Amplification of the downstream region of *art9* (*Hin*dIII)
28-L-F<br>28-L-R	aaagacataatcgatAAGCTTgcaaccagtgtaatgatgatcg<br>accaacagctaggcacaattgaatgtgataagagccagaag	Amplification of the upstream region of *art28* (*Hin*dIII)
28-R-F<br>28-R-R	cttctggctcttatcacattcaattgtgcctagctgttggt<br>taaactaccgcattaAAGCTTgggattcagtaagtgacgagat	Amplification of the downstream region of *art28* (*Hin*dIII)
(-1)-F<br>(-1)-R	aaagacataatcgatAAGCTTtgggctacacacgtgctac<br>taaactaccgcattaAAGCTTggtgtgtacaaggcccgg	Amplification of a single homologous arm of gene *art(-1)* (*Hin*dIII)
(-1)ver-F<br>(-1)ver-R	ggggagaaaacatagggggt<br>cttgtaggcacacggtttcaggat	PCR confirmation of genotype of mutant Δ*orf(-1)*
(+1)-F<br>(+1)-R	aaagacataatcgatAAGCTTaaatcgctcaggacatccgc<br>taaactaccgcattaAAGCTTttcggaagtcgggacgaact	Amplification of a single homologous arm of gene *art(-1)* (*Hin*dIII)
(+1)ver-F	gagcgcattgttagatttc	PCR confirmation of genotype of mutant

(+1)ver-R	aacgcatgataataccgct	Δ*orf(+1)*
11MT-L-F	cactatggcgtgctGCTAGCcagggttatgacctggatt	Amplification of the upstream region of His$_{735}$ codon of gene *art11*
11MT-L-R	ctgcataatattttttgtGGCggcaagcacattggtcgcaatggc	
11MT-R-F	gccattgcgaccaatgtgcttgccGCCacaaaaaatattatgcag	Amplification of the downstream region of His$_{735}$ codon of gene *art11*
11MT-R-R	tcaacgcatatagcGCTAGCcgttgctgaggatccatat	
13MT-L-F	cactatggcgtgctGCTAGCggctatctgagaccatcgct	Amplification of the upstream region of His$_{3320}$ codon of gene *art13*
13MT-L-R	ctcccgtaatactggggtagcGGCaaggacattactggcaatgac	
13MT-R-F	gtcattgccagtaatgtccttGCCgctaccccagtattacgggag	Amplification of the downstream region of His$_{3320}$ codon of gene *art13*
13MT-R-R	tcaacgcatatagcGCTAGCggtgtataacctgcatctgc	
14MT-L-F	cactatggcgtgctGCTAGCctaagaccgacaccatcct	Amplification of the upstream region of His$_{1602}$ codon of gene *art14*
14MT-L-R	gtggcgaatggcttgggtagcGGCgagtacattactggcgataag	
14MT-R-F	cttatcgccagtaatgtactcGCCgctacccaagccattcgccac	Amplification of the downstream region of His$_{1602}$ codon of gene *art14*
14MT-R-R	tcaacgcatatagcGCTAGCgagcggatcaaaggcgtt	
17MT-L-F	cactatggcgtgctGCTAGCtatcagacacccagacaccg	Amplification of the upstream region of His$_{255}$ codon of gene *art17*
17MT-L-R	gctttatatctcgggtGGCggcgagcacatttgccgccacc	
17MT-R-F	ggtggcggcaaatgtgctcgccGCCacccgagatataaagc	Amplification of the downstream region of His$_{255}$ codon of gene *art17*
17MT-R-R	tcaacgcatatagcGCTAGCtaggtagcttccatacccg	
28-9-F	tgccgcgcggcagcCATATGggagaacttgaagtcat	Amplification of *art9* (*Nde*I and *Bam*HI)
28-9-R	cggagctcgaattcGGATCCtcatgcttcatctaccattcg	
28-10-F	tgccgcgcggcagcCATATGaagcatgatcgaagggt	Amplification of *art10acp* (*Nde*I and *Bam*HI)
28-10-R	cggagctcgaattcGGATCCttattcctctgccgagtatagtt	
28-28-F	tgccgcgcggcagcCATATGaattcttctggctcttat	Amplification of *art28* (*Nde*I and *Bam*HI)
28-28-R	cggagctcgaattcGGATCCttatacagtttttcaccaacag	

*The designed restriction site in each primer is capitalized and underlined.

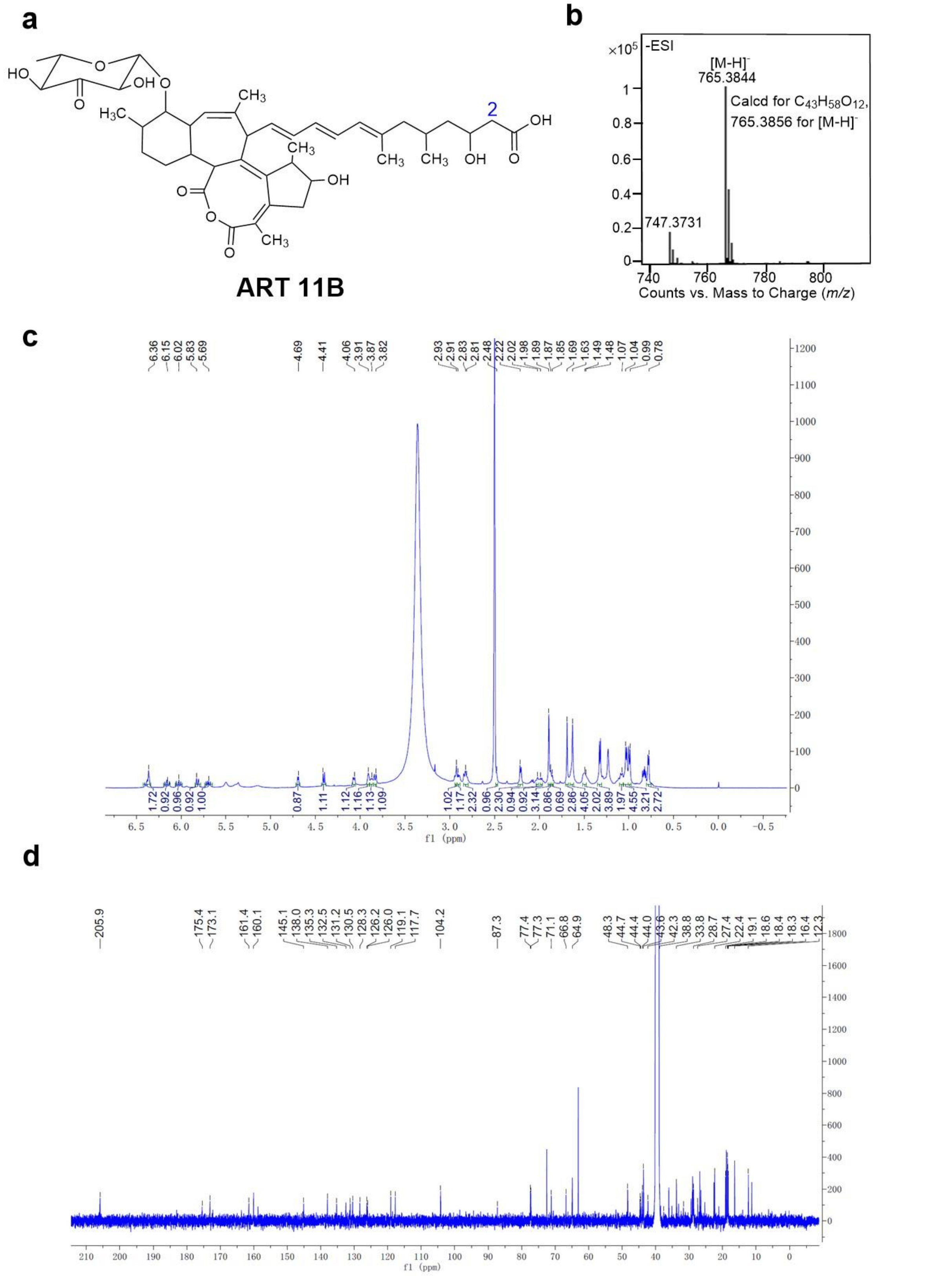

Supplementary Fig. 1. Spectral data of ART 11B. (**a**) Structure of ART 11B. (**b**) HR-ESI-MS spectrum of ART 11B. (**c**) ^{1}H NMR spectrum of ART 11B in DMSO-$_{d6}$. (**d**) ^{13}C NMR spectrum of ART 11B in DMSO-$_{d6}$.

e

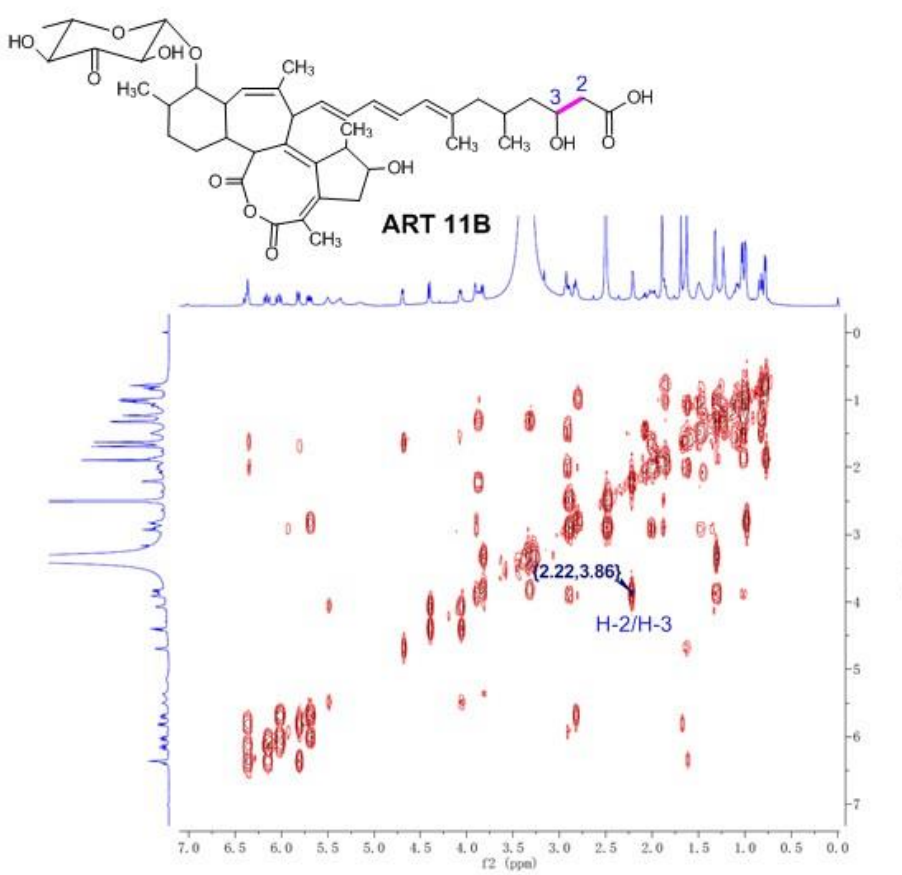

f

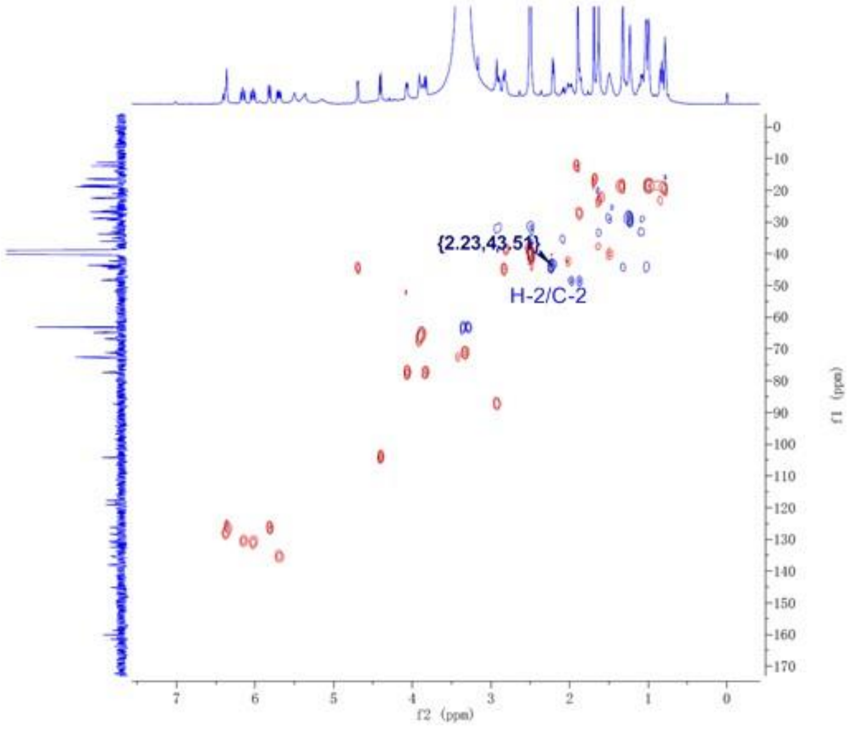

Supplementary Fig. 1. Spectral data of ART 11B. **(e)** COSY spectrum of ART 11B in DMSO-$_{d6}$. The key ^{1}H-^{1}H COSY correlations are marked with pink bold lines and denoted at the spectrum. **(f)** HSQC spectrum of ART 11B in DMSO-$_{d6}$. The HSQC correlation of C-2 is denoted.

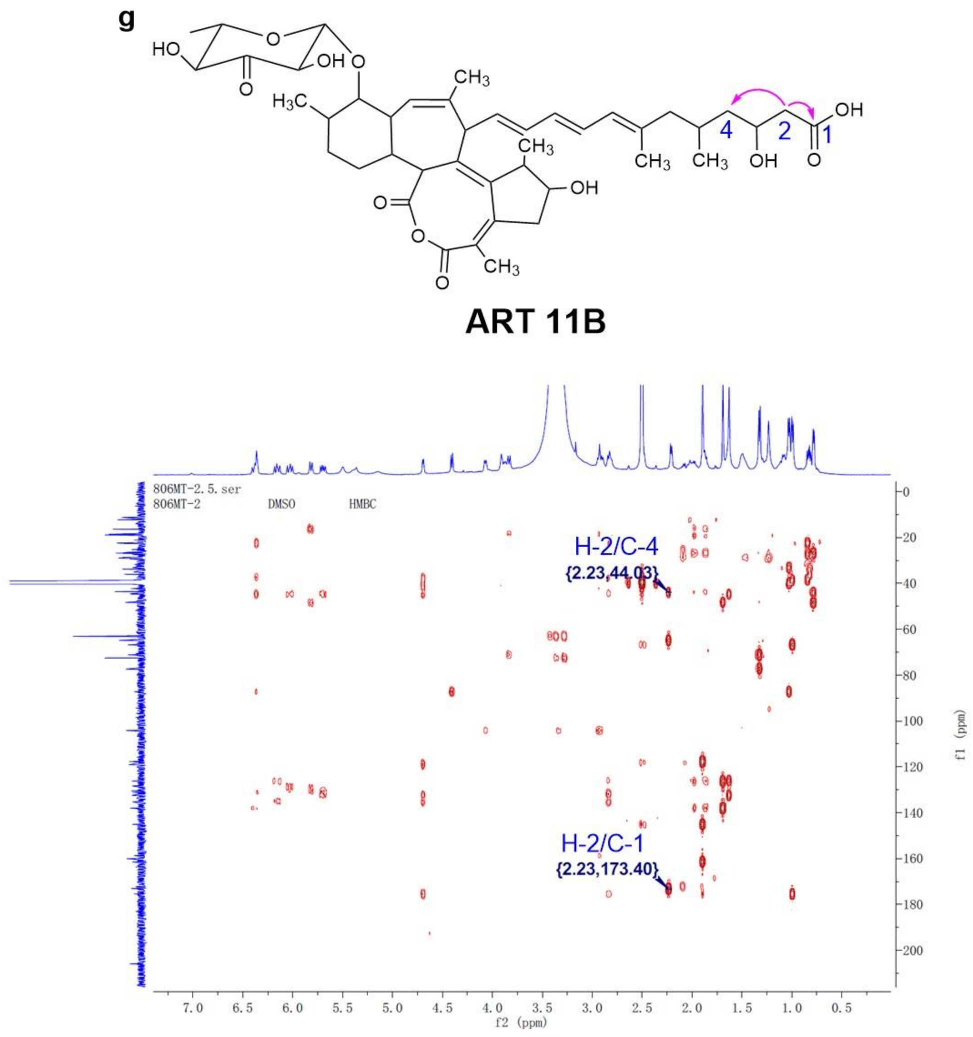

Supplementary Fig. 1. Spectral data of ART 11B. **(g)** HMBC spectrum of ART 11B in DMSO-$_{d6}$. The key HMBC correlations are marked with pink arrows and denoted at the spectrum.

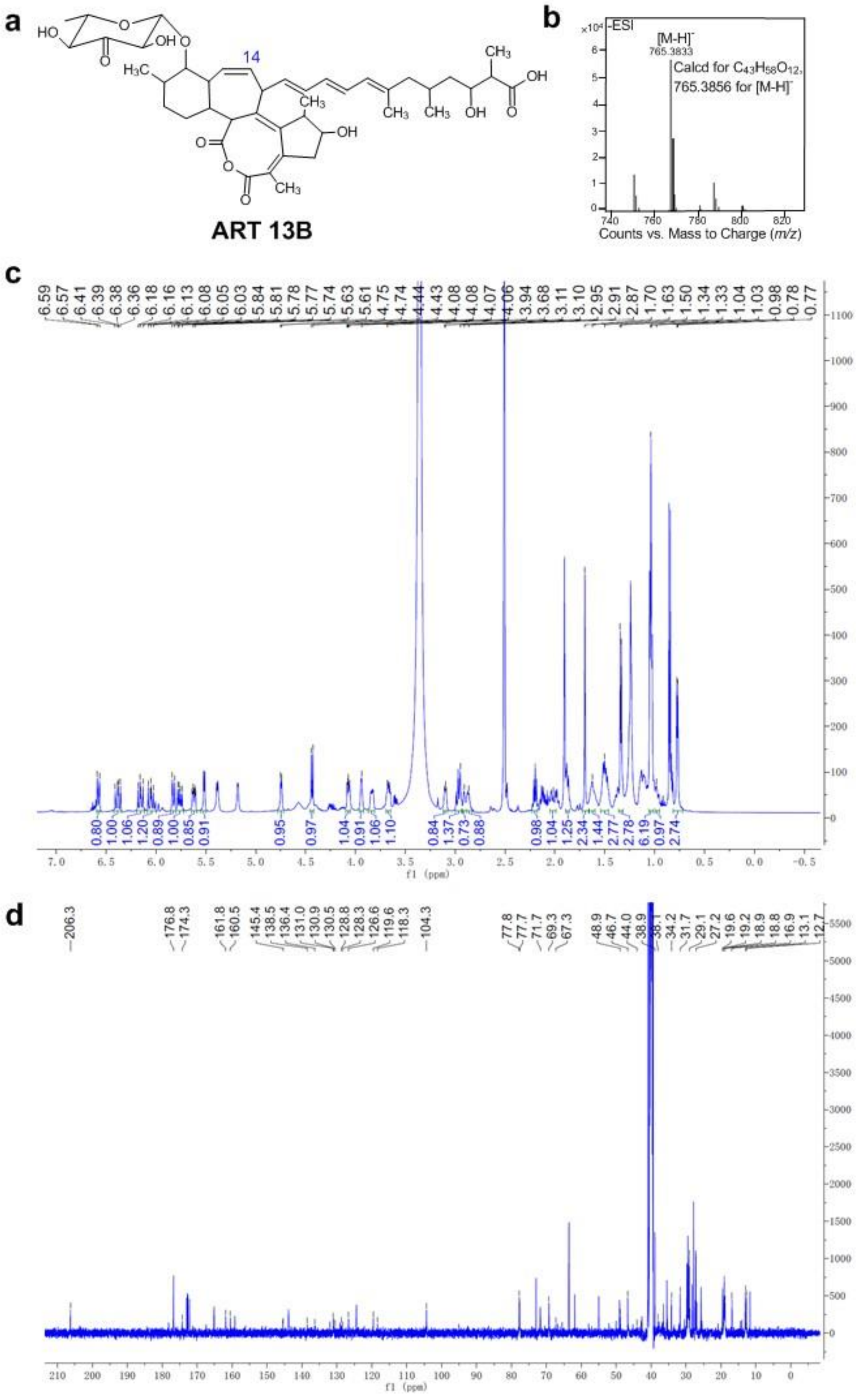

Supplementary Fig. 2. Spectral data of ART 13B. (**a**) Structure of ART 13B. (**b**) HR-ESI-MS spectrum of ART 13B. (**c**) ^{1}H NMR spectrum of ART 13B in DMSO-$_{d6}$. (**d**) ^{13}C NMR spectrum of ART 13B in DMSO-$_{d6}$.

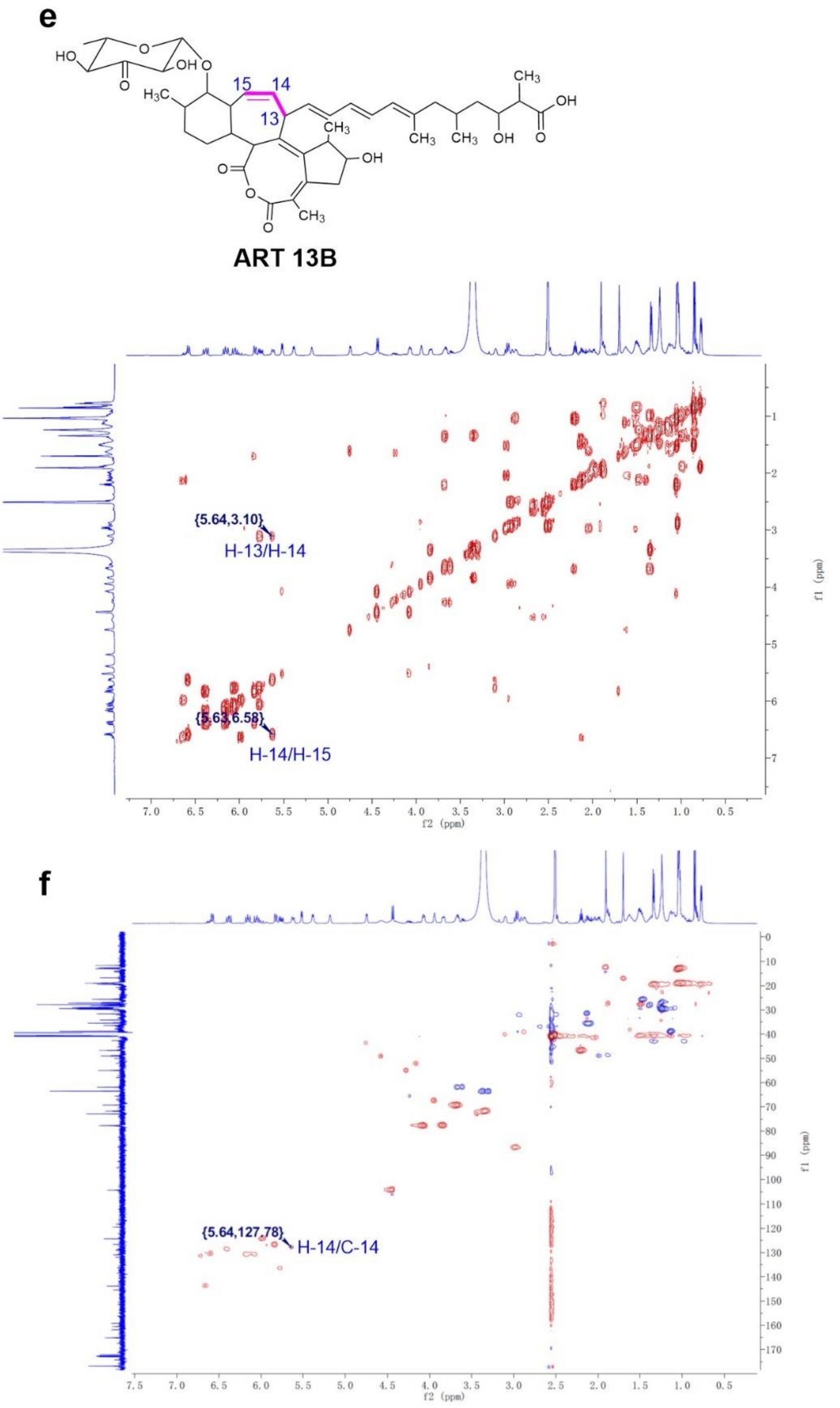

Supplementary Fig. 2. Spectral data of ART 13B. **(e)** COSY spectrum of ART 13B in DMSO-$_{d6}$. The key ^{1}H-^{1}H COSY correlations are marked with pink bold lines and denoted at the spectrum. **(f)** HSQC spectrum of ART 13B in DMSO-$_{d6}$. The HSQC correlation of C-14 is denoted.

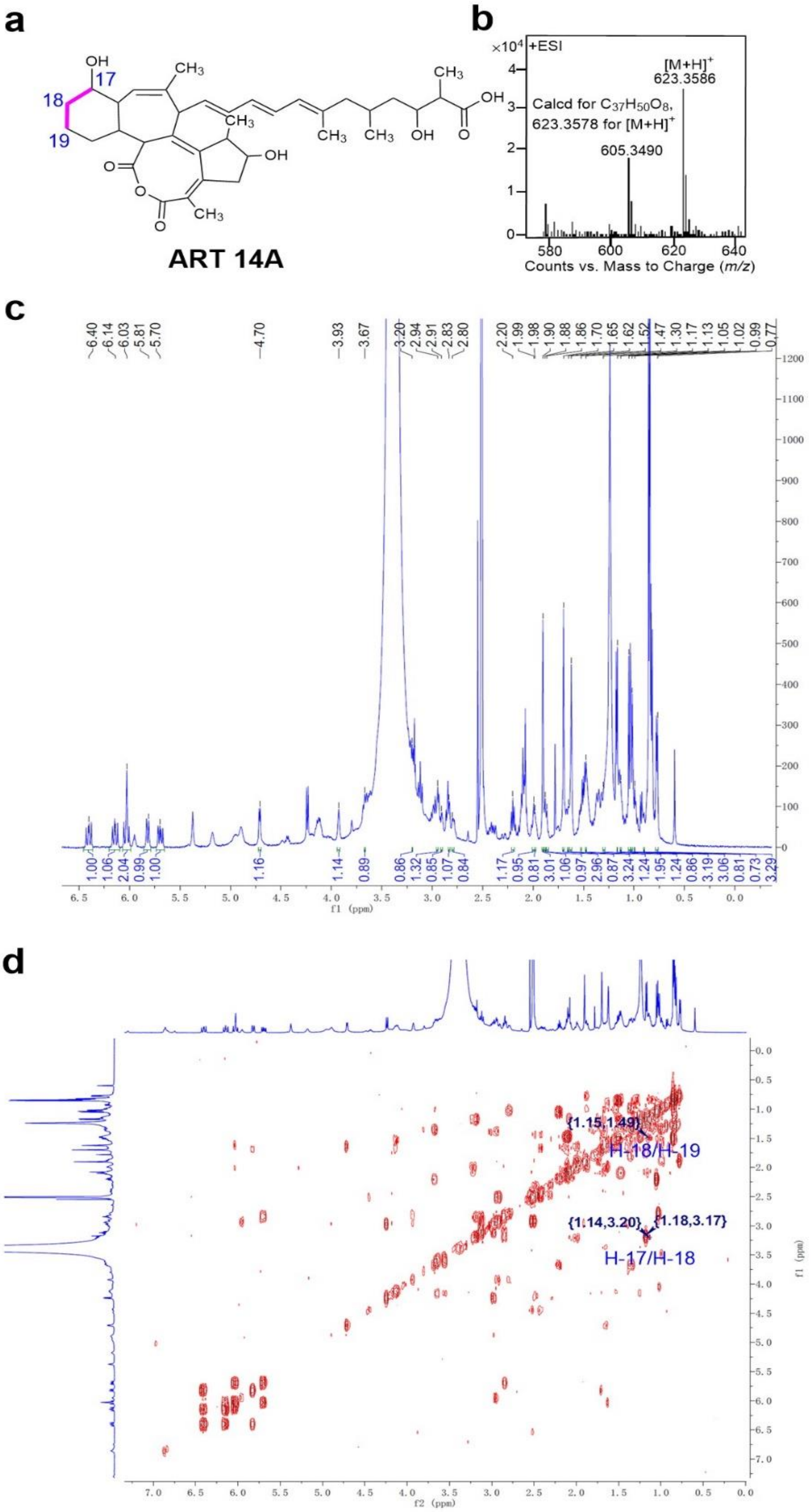

Supplementary Fig. 3. Spectral data of ART 14A. (**a**) Structure of ART 14A. (**b**) HR-ESI-MS spectrum of ART 14A. (**c**) ^{1}H NMR spectrum of ART 14A in DMSO-$_{d6}$. (**d**) COSY spectrum of ART 14A in DMSO-$_{d6}$. The key COSY correlations are marked with pink bold lines in ART 14A structure and denoted at the spectrum.

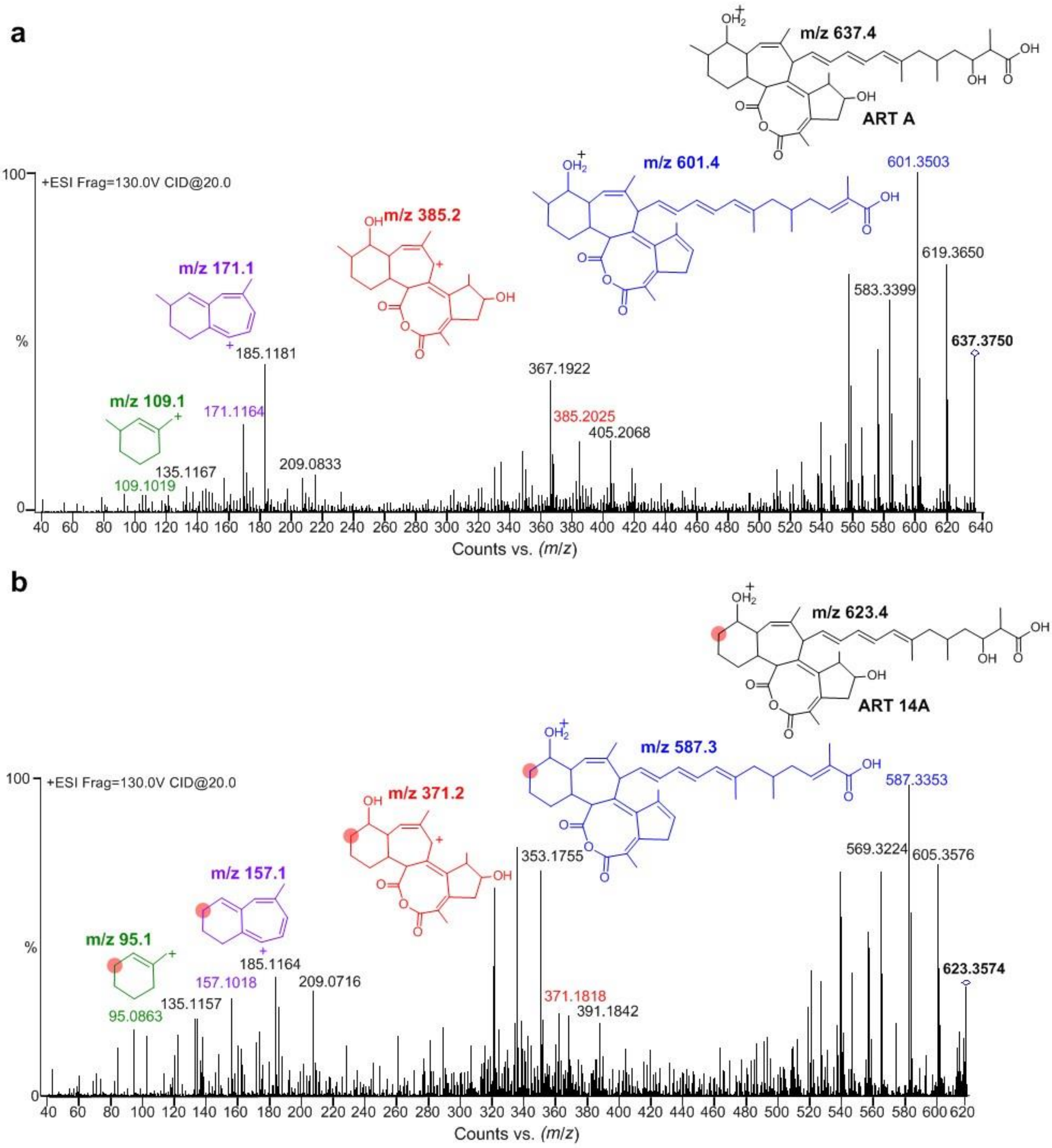

Supplementary Fig. 4. Tandem MS analysis of ART A and ART 14A. Fragmentation patterns of ART A (**a**) and ART 14A (**b**).

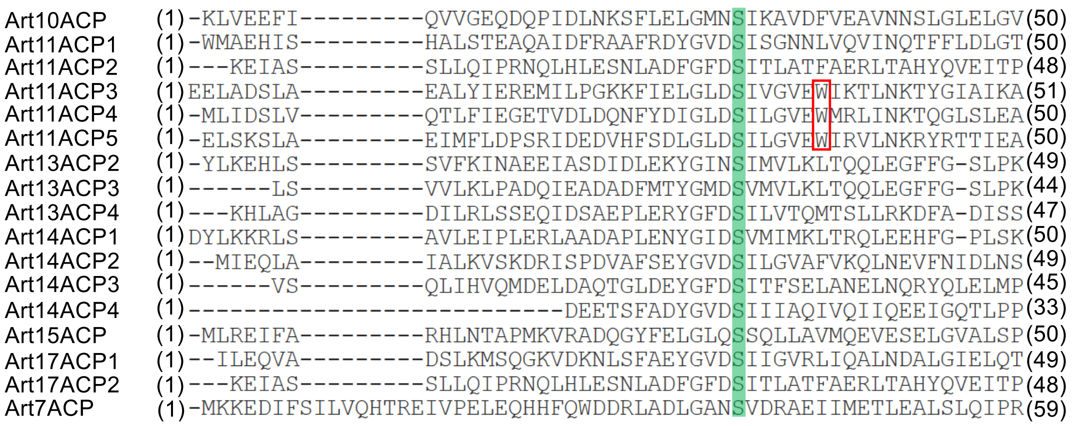

Supplementary Fig. 5. Sequence alignment of the ACP domains of different ART PKS modules. The conserved serine residue for the attachment of 4′-Ppant arm is colored green. The critical tryptophan residues in Art11ACP3, Art11ACP4, and Art11ACP5 interacting with HMGS in β-branching system are highlighted with a red box.

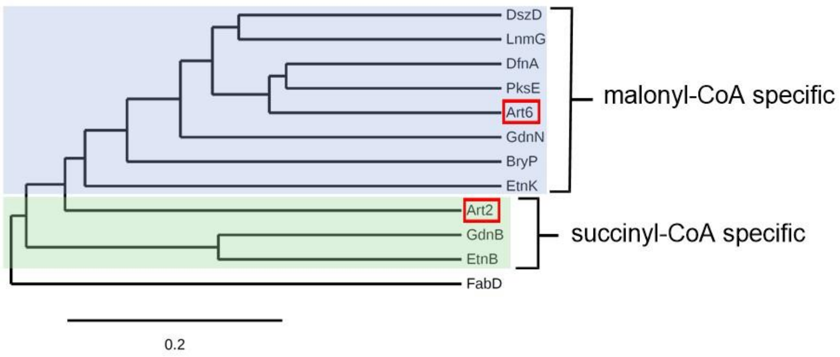

Supplementary Fig. 6. Phylogenetic analysis of acyltransferases from different *trans*-AT PKSs. Sequences from the following natural products were selected: aurantinin (*Bacillus subtilis* fmb60) (Art2, WP_069149215.1; Art6, WP_069149219.1); bacillaene (*Bacillus amyloliquefaciens*) (PksE, WP_012117591.1); bryostatin (*Candidatus Endobugula* sertul) (BryP, ABM63531.1); disorazol (*Sorangium cellulosum*) (DszD, AAY32968.1); etnangien (*Sorangium cellulosum* So ce56) (EtnB, WP_012235817.1; EtnK, CAN93354.1); gladiolin (*Burkholderia gladioli*) (GbnB, WP_052409166.1; GnbN, WP_105859602.1); leinamycin (*Streptomyces atroolivaceus*) (LnmG, AAN85520.1); difficidin (*Bacillus velezensis* FZB42) (DfnA, CAG23974.1). Outgroup is the *Yarrowia lipolytica* acyltransferase (FabD, XP_504110.1). Art2 and Art6 fall into two different clades and were highlighted with red boxes.

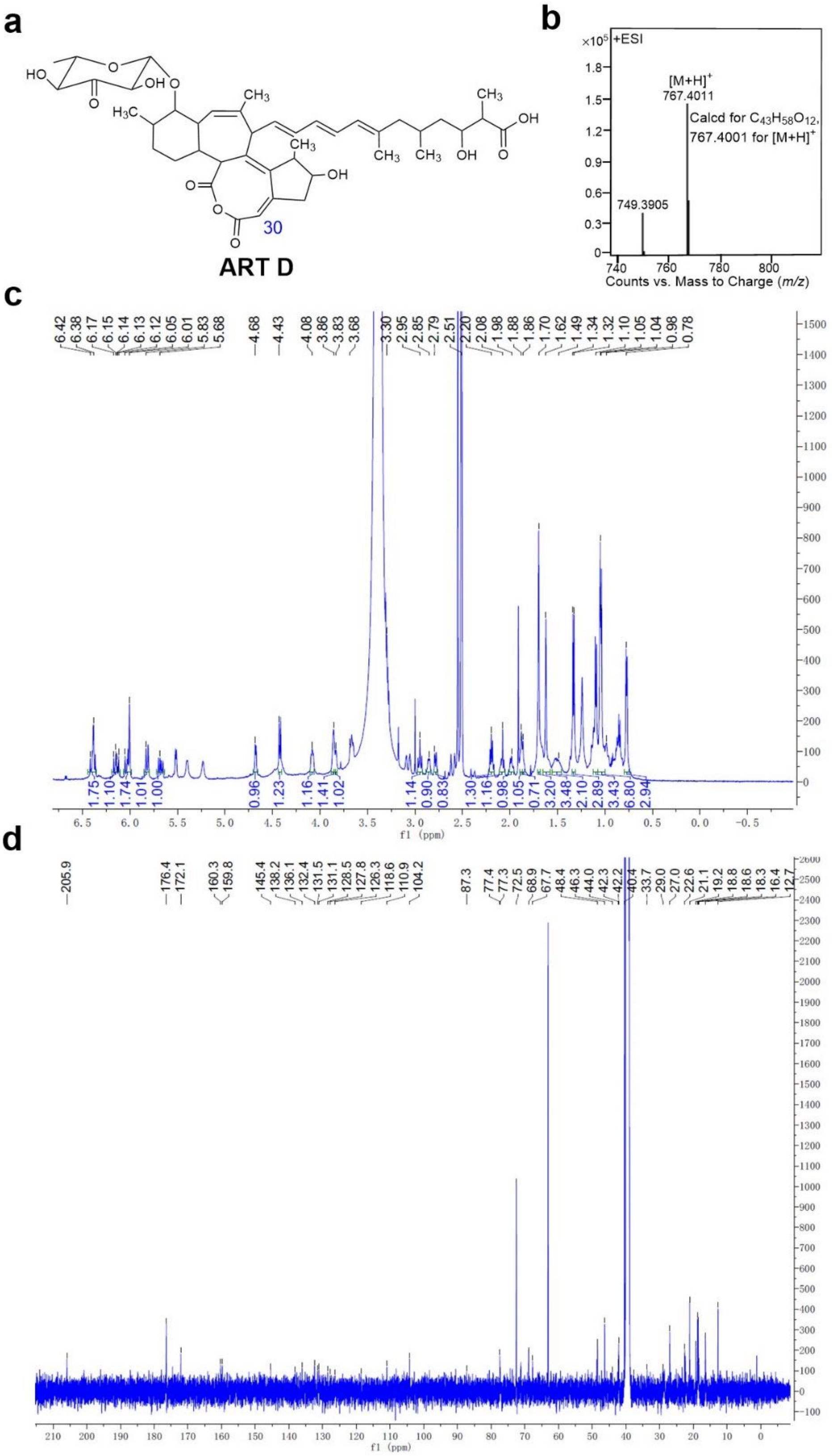

Supplementary Fig. 7. Spectral data of ART D. (**a**) Structure of ART D. (**b**) HR-ESI-MS spectrum of ART D. (**c**) ^{1}H NMR spectrum of ART D in DMSO-d_6. (**d**) ^{13}C NMR spectrum of ART D in DMSO-d_6.

e

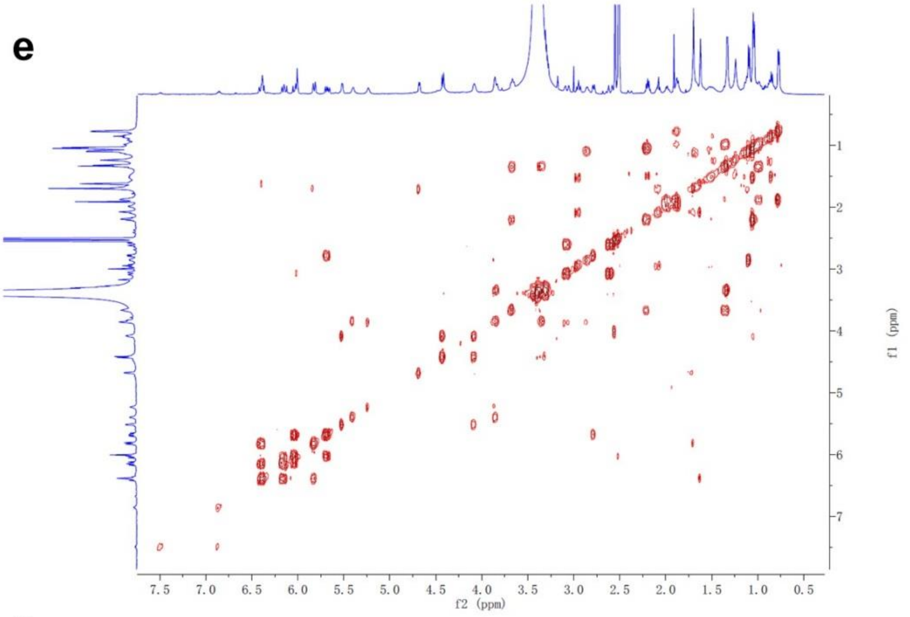

f

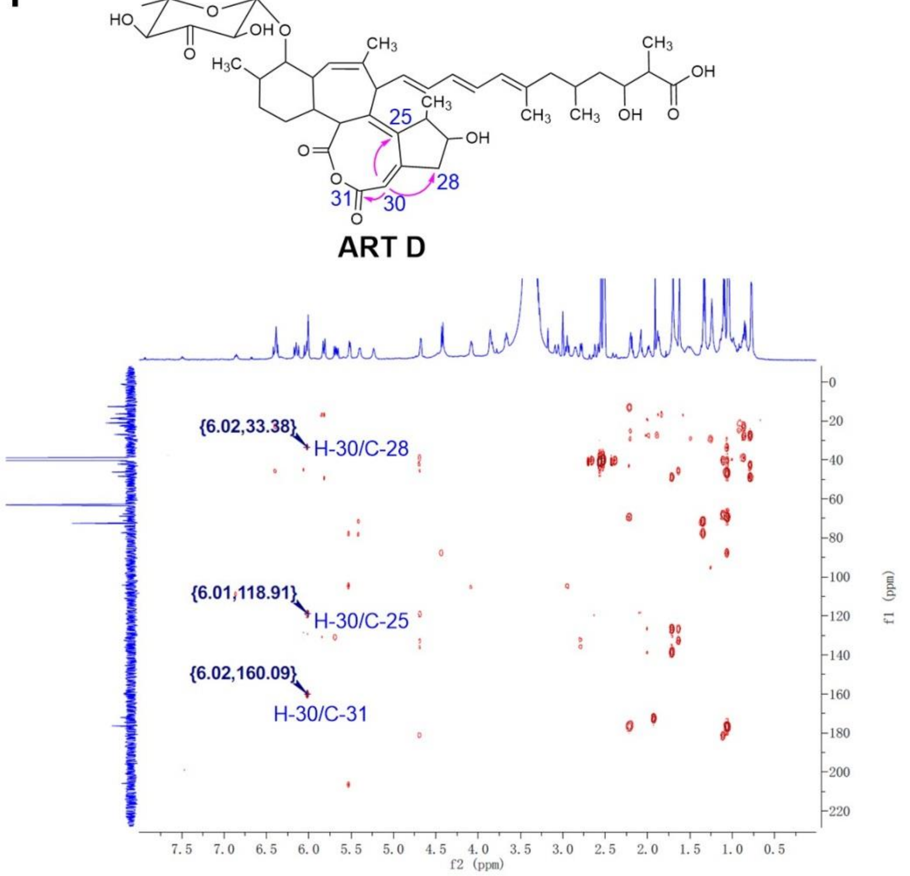

Supplementary Fig. 7. Spectral data of ART D. (**e**) COSY spectrum of ART D in DMSO-$_{d6}$. (**f**) HMBC spectrum of ART D in DMSO-$_{d6}$. The key HMBC correlations are marked with pink arrows and denoted at the spectrum.

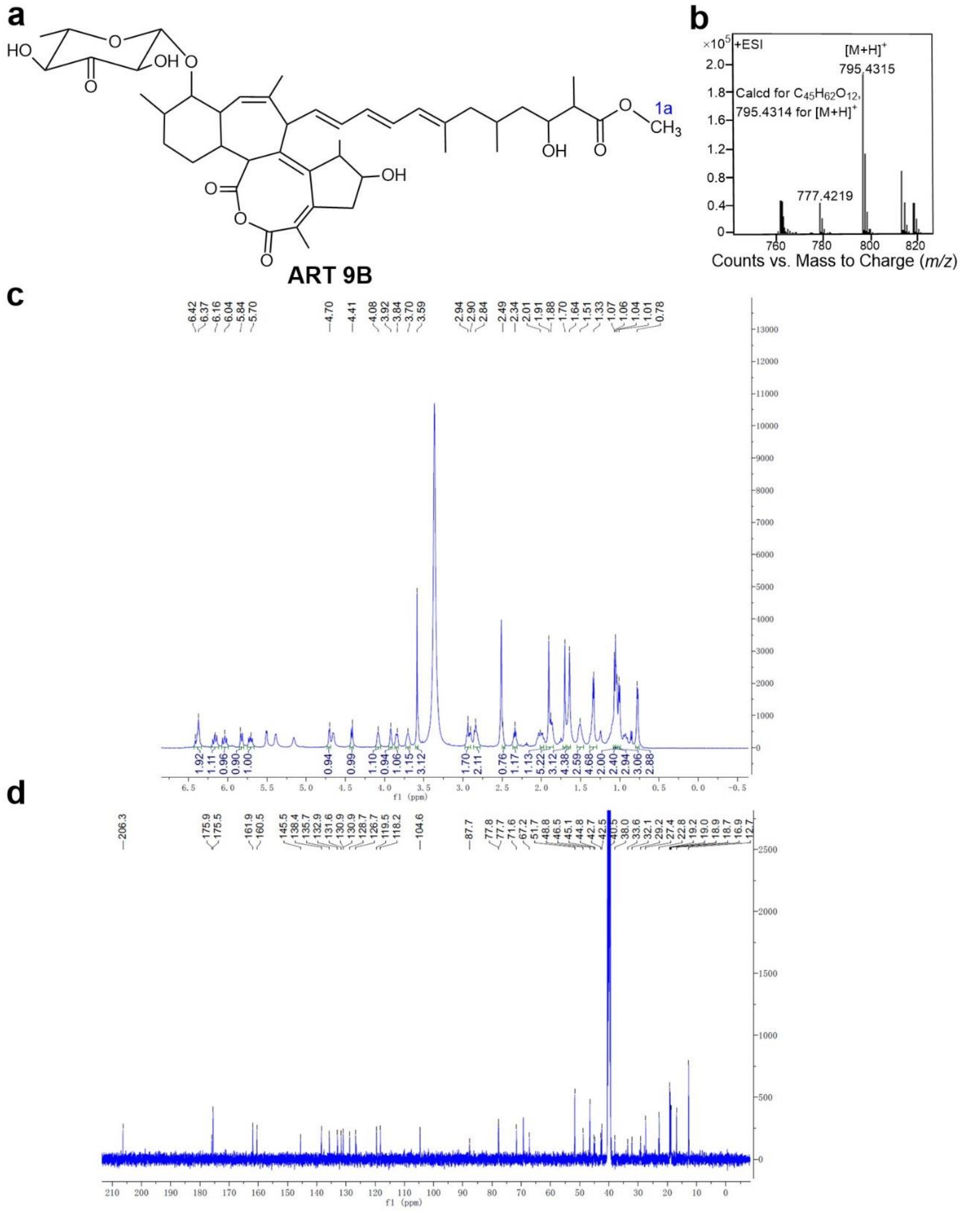

Supplementary Fig. 8. Spectral data of ART 9B. (**a**) Structure of ART 9B. (**b**) HR-ESI-MS spectrum of ART 9B. (**c**) **^{1}**H NMR spectrum of ART 9B in DMSO-*d6*. (**d**) **^{13}**C NMR spectrum of ART 9B in DMSO-*d6*.

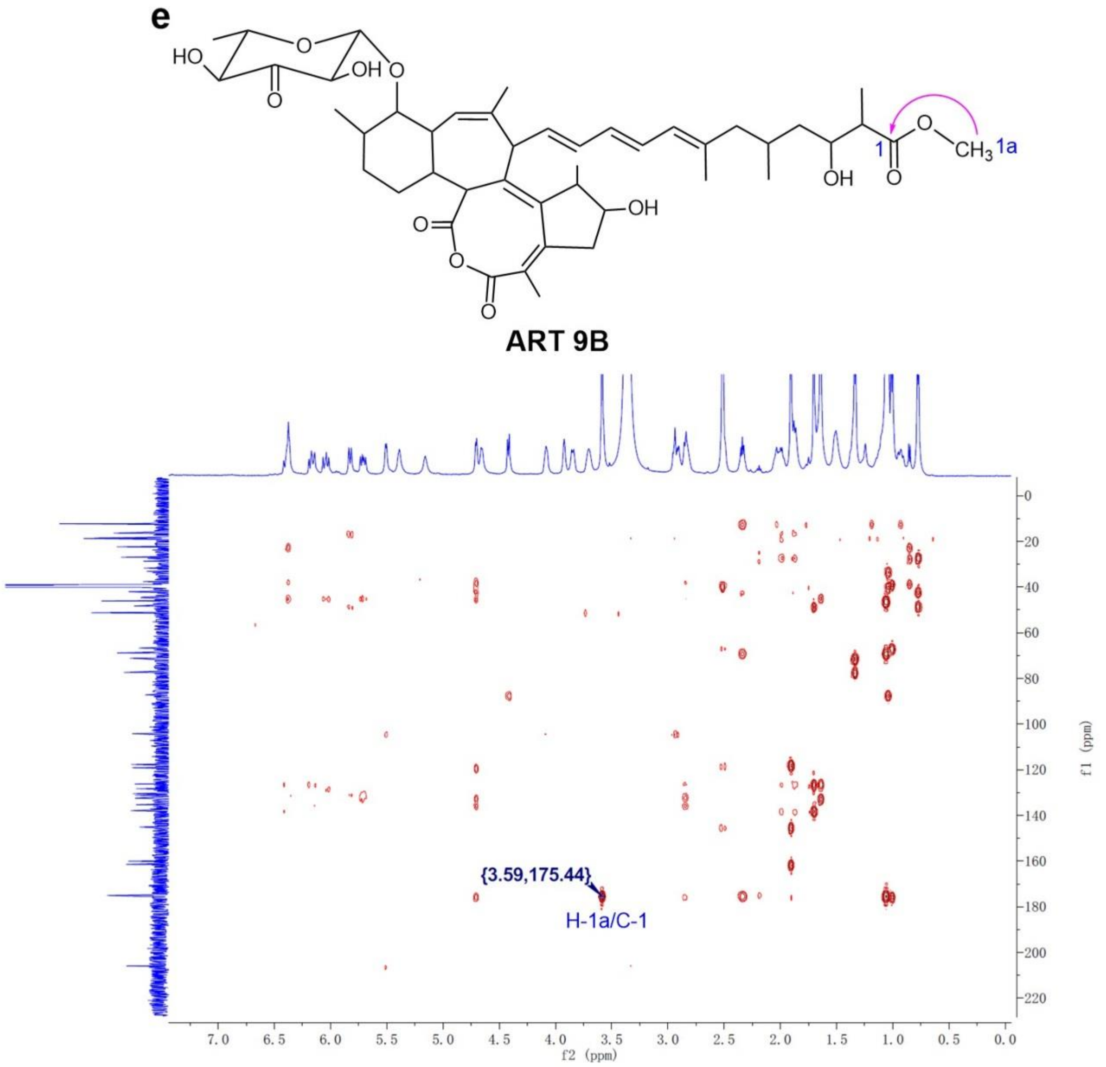

Supplementary Fig. 8. Spectral data of ART 9B. (**e**) HMBC spectrum of ART 9B in DMSO-$_{d6}$. The key HMBC correlations are marked with pink arrows and denoted at the spectrum.

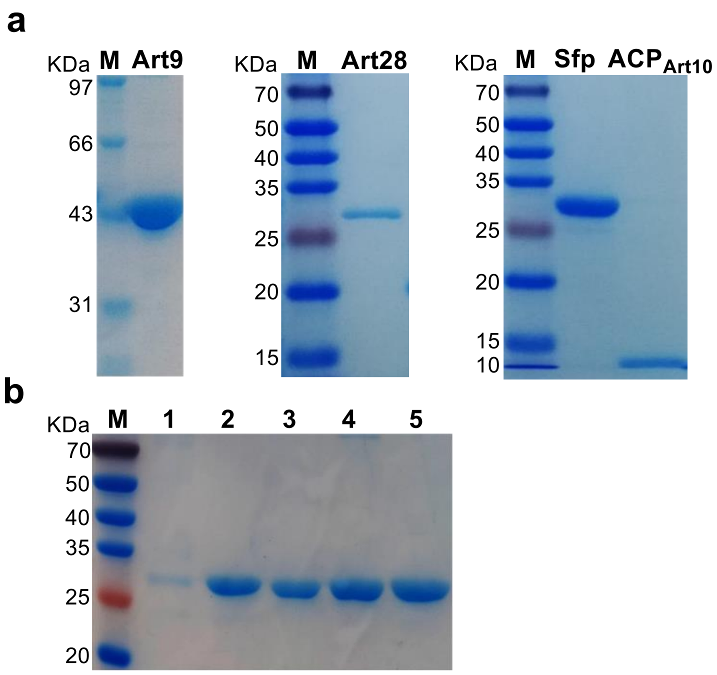

Supplementary Fig. 9. SDS-PAGE analysis of purified proteins. (**a**) SDS-PAGE analysis of *N*-His_6 tagged Art9, Art28, Sfp, and ACP_{Art10}. (**b**) SDS-PAGE analysis of five *N*-His_6-tagged Art28-like proteins. Lane 1, Clp905 from *Clostridium papyrosolvens* DSM2782； Lane 2, Pas675 from *Paenibacillus* sp. Mc5Re; Lane 3, Brf335 from *Brevibacillus formosus*; Lane 4, Thd235 from *Thermoactinomyces daqus* H-18; Lane 5, Bac390 from *Bacillus cereus* BAG5X1-1. Lane M, protein marker. Each experiment was repeated independently at least three times. Source data are provided as a Source Data file.

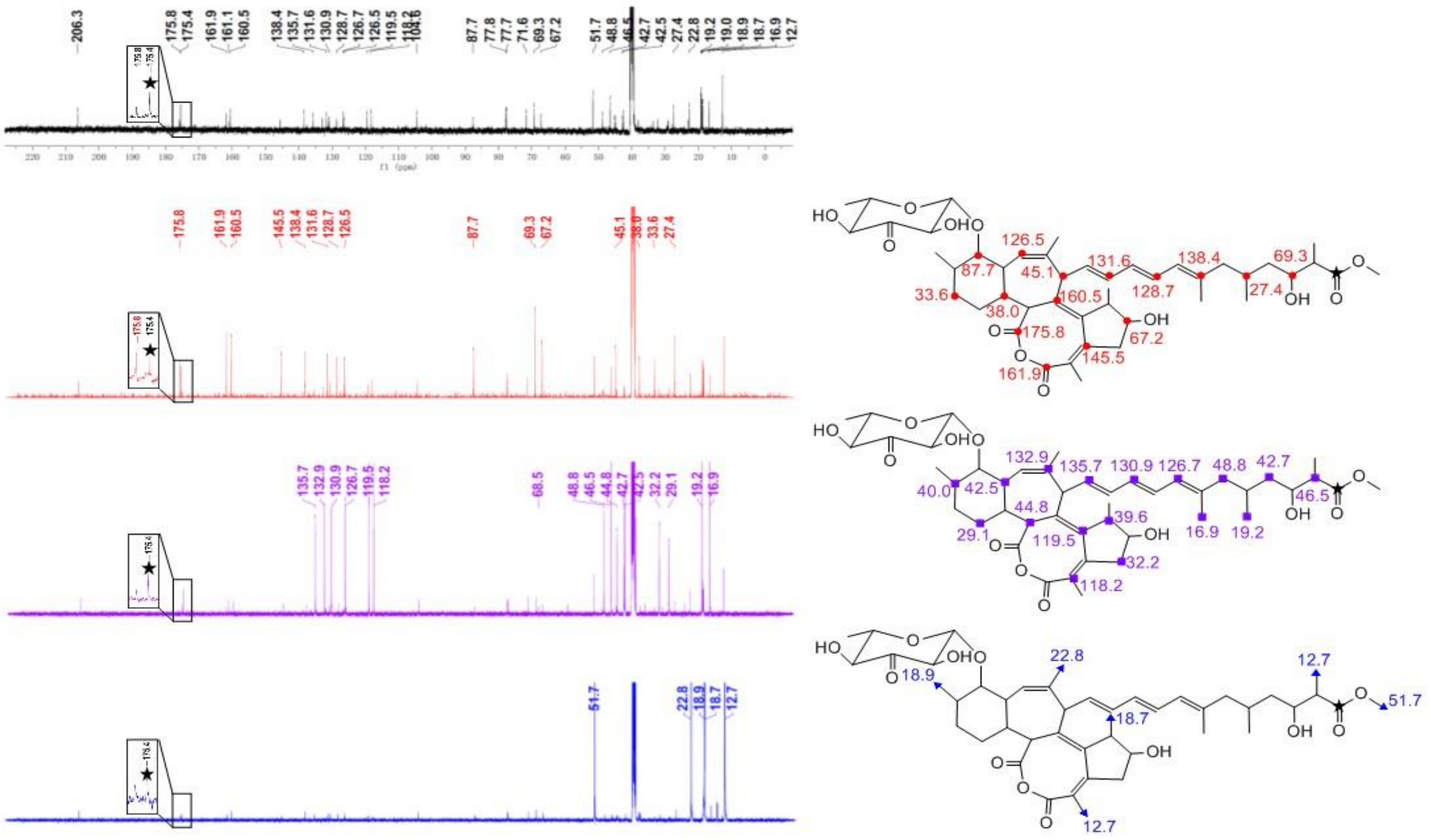

Supplementary Fig. 10. The ^{13}C NMR spectra of ART 9B labeled with diverse ^{13}C labeled precursors. Natural abundance (black); [1-^{13}C] sodium acetate (red); [2-^{13}C] sodium acetate (purple); [^{13}C-methyl]-methionine (blue)**.** Black stars denote the signal of C-1 (δ_C=175.4) in ART 9B**.**

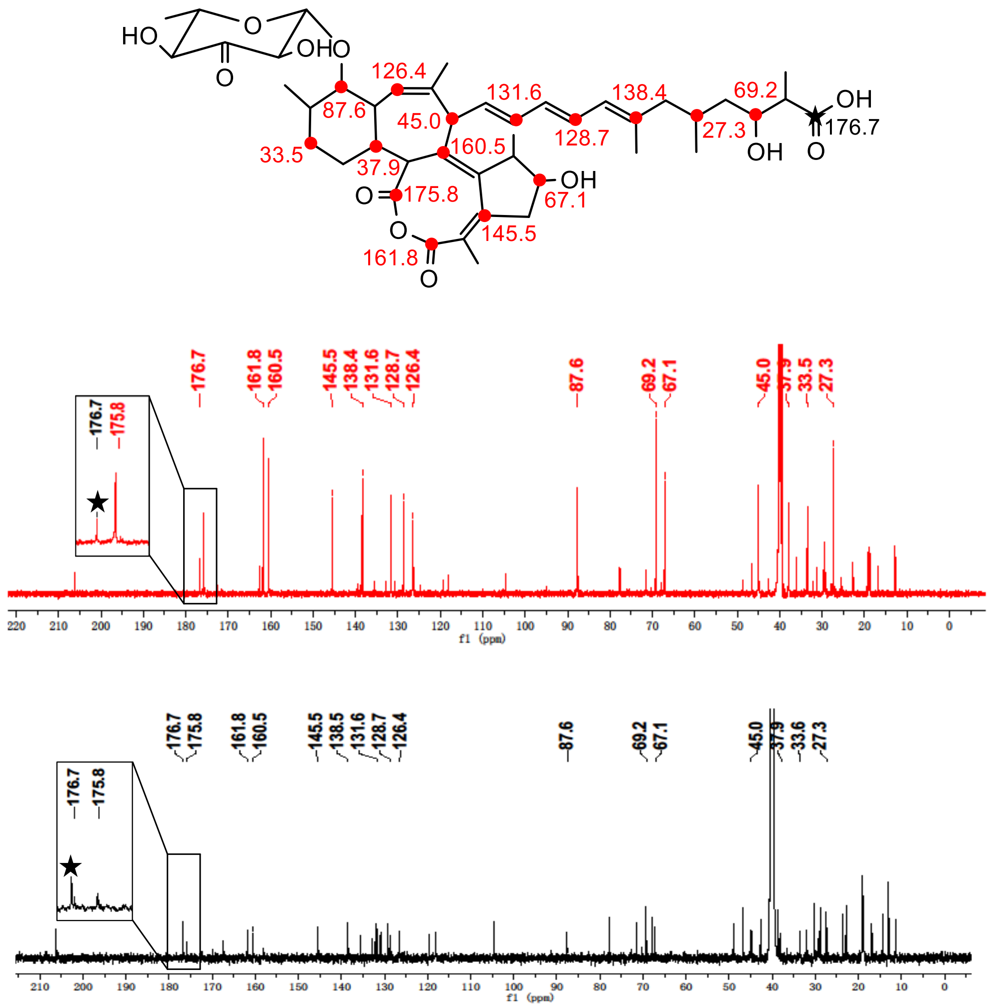

Supplementary Fig. 11. The ^{13}C NMR spectra of ART B labeled with [1-^{13}C] sodium acetate. Natural abundance (black); [1-^{13}C] sodium acetate (red)**.** Black stars denote the signal of C-1 (δ_C=176.7) in ART B**.**

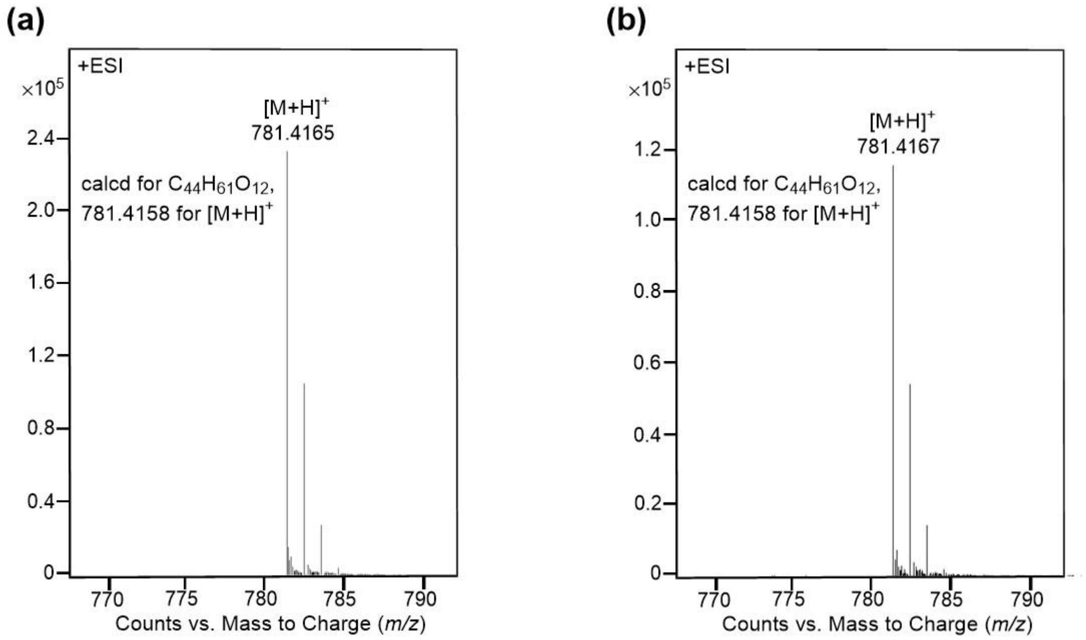

Supplementary Fig. 12. HRMS analysis of the production of ART B in *B. subtilis* Δ*art28*/*Bc-bioC* (**a**) and in the Art9 assay (**b**).

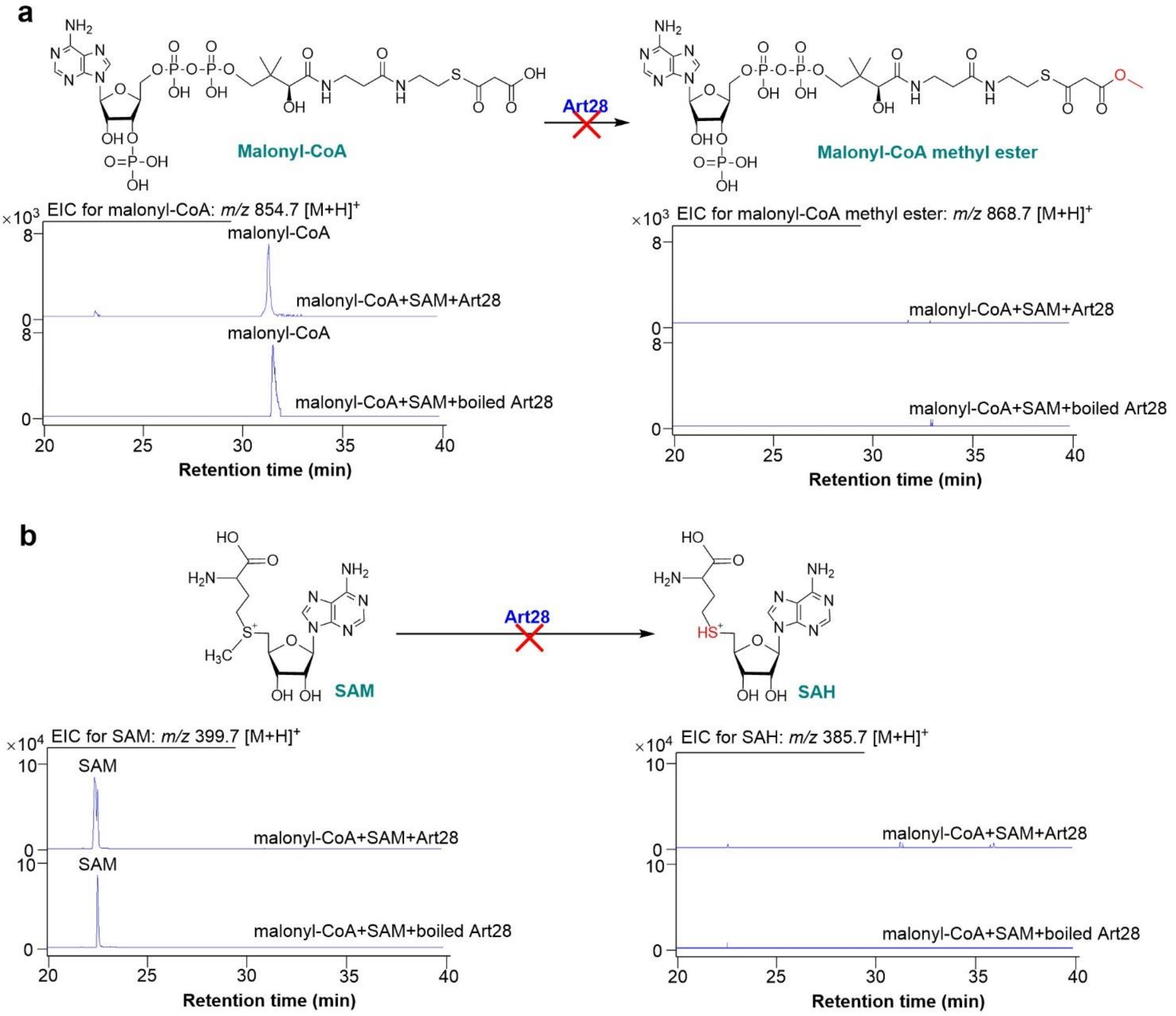

Supplementary Fig. 13. LC-MS analysis of the *in vitro* assays of Art28 using malonyl-CoA as a substrate. (**a**) The production of malonyl-CoA methyl ester (*m/z* $[M+H]^+ = 868.7$) was analyzed by LC-MS. (**b**) The generation of SAH (*m/z* $[M+H]^+ = 385.7$) was analyzed by LC-MS.

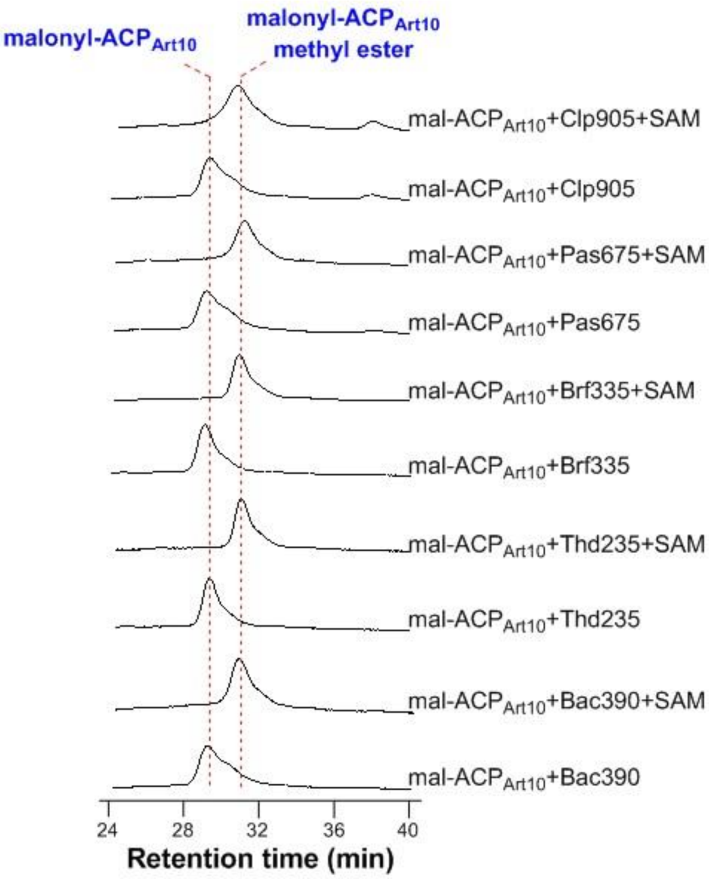

Supplementary Fig. 14. HPLC analysis of the malonyl-ACP *O*-MT activity of the five Art28 homologues.

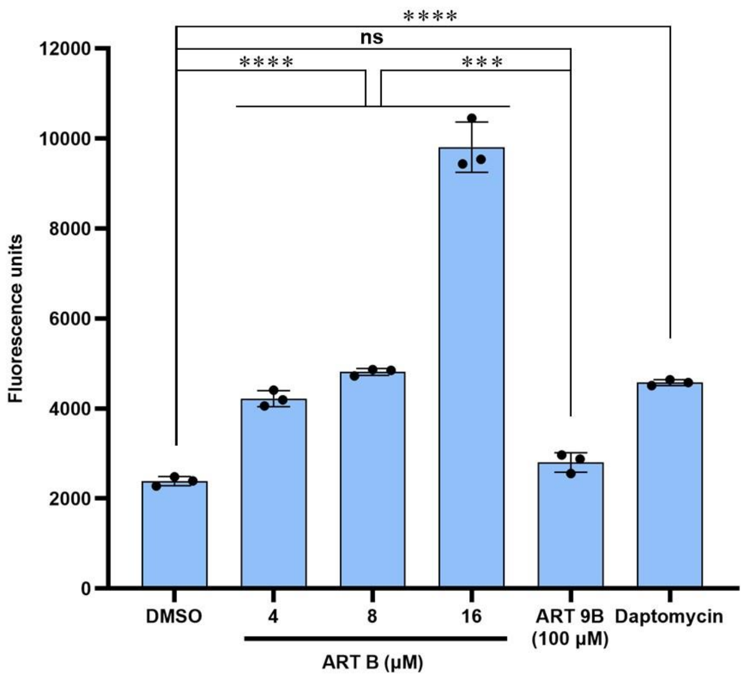

Supplementary Fig. 15. The cell membrane permeability was assessed using propidium iodide after exposure of *Staphylococcus aureus* ATCC 6538 to ART B or ART 9B. Data are presented as the means ± standard deviation (SD), n=3 independent experiments. Statistical significance was calculated using a two-tailed one-way ANOVA followed by Tukey post-hoc test and *P* values are indicated (ns, $P > 0.05$, * $P \leq 0.05$, ** $P \leq 0.01$, *** $P \leq 0.001$, **** $P \leq 0.0001$). Source data are provided as a Source Data file.

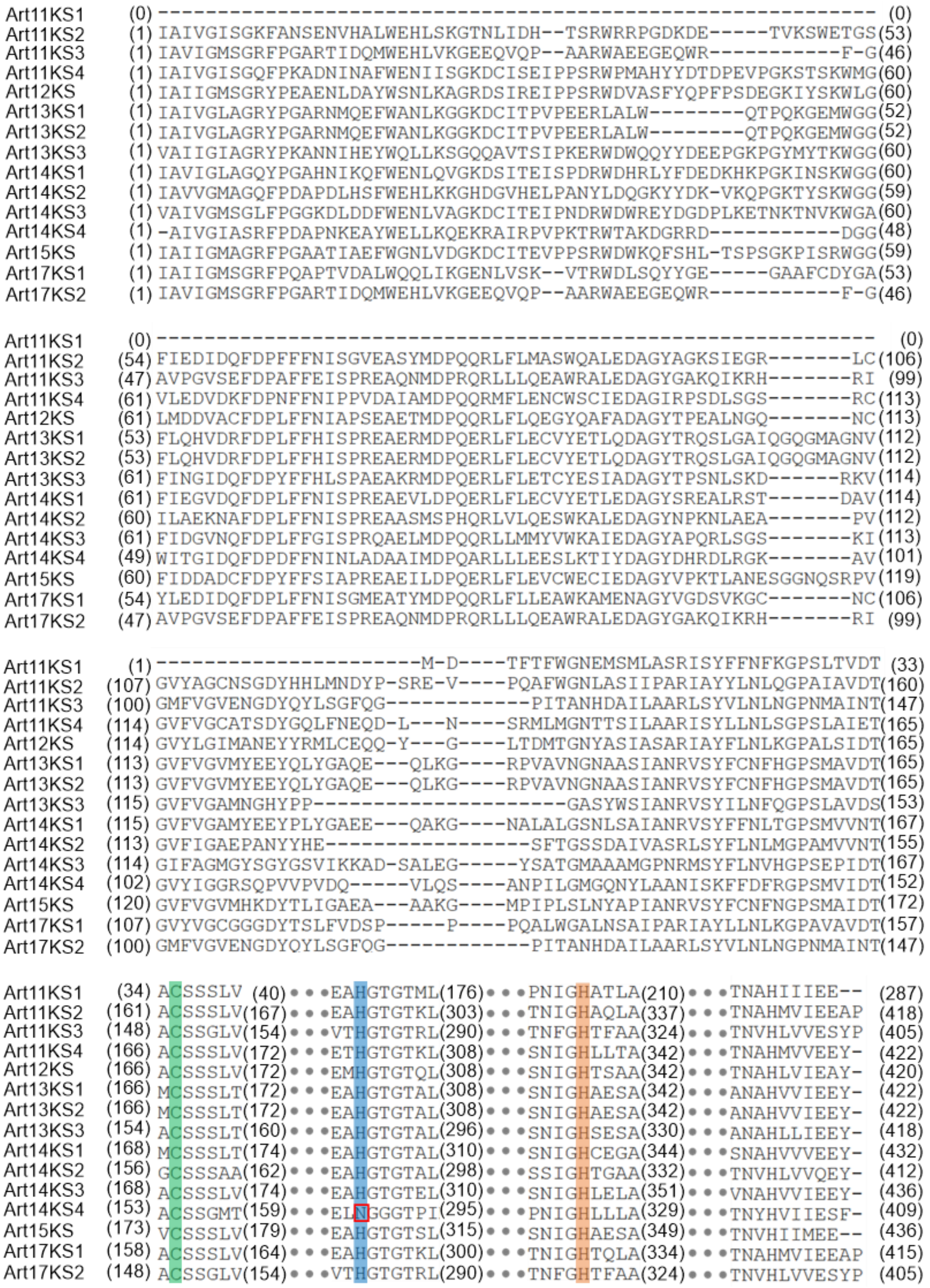

Supplementary Fig. 16. Sequence alignment of the KS domains of different ART PKS modules. The Art11KS1 domain was incomplete due to lack of about 130-aa *N*-terminal sequence. The conserved Cys and two His residues of KS domains[4] are highlighted in green, blue and orange, respectively. The mutation of the first conserved His residue to Asn in Art14KS4 is indicated with a red box.

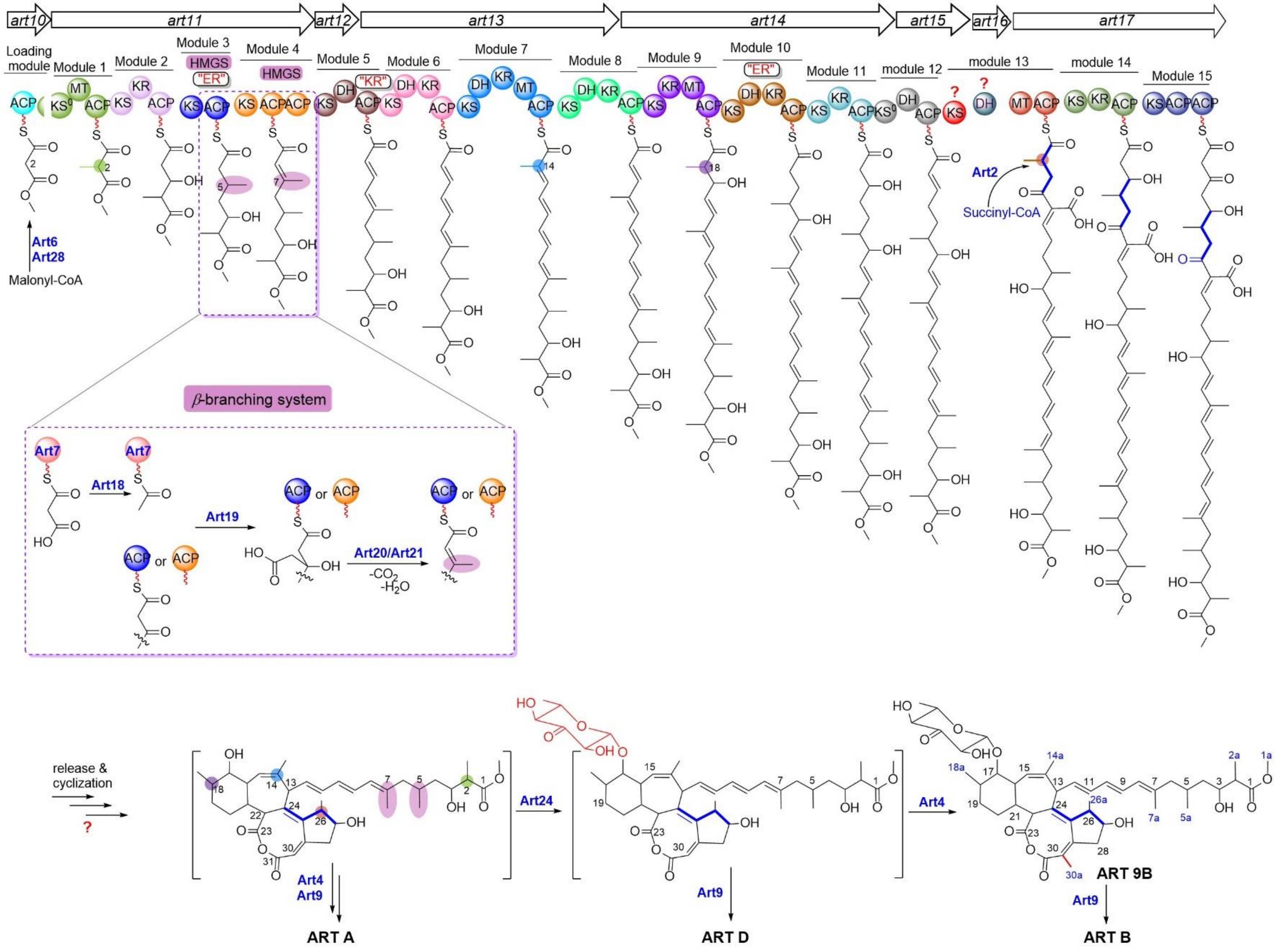

Supplementary Fig. 17. The proposed biosynthetic pathway of ARTs based on the one-polyketide-chain assembly model.

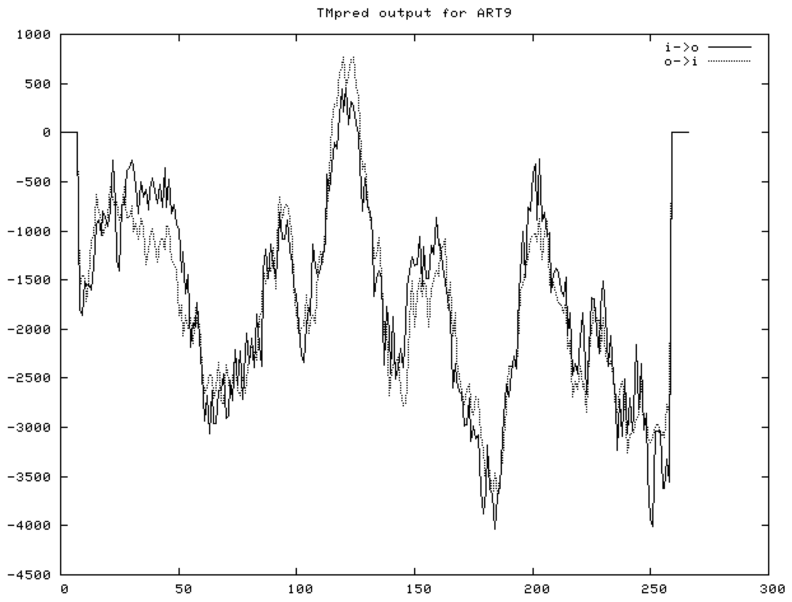

Supplementary Fig. 18. Transmembrane region analysis of Art9. Twenty amino acids (residue 111-130) of Art9 were predicted as the transmembrane helix with N-terminus outside. i->o: inside to outside; o->i: outside to inside.

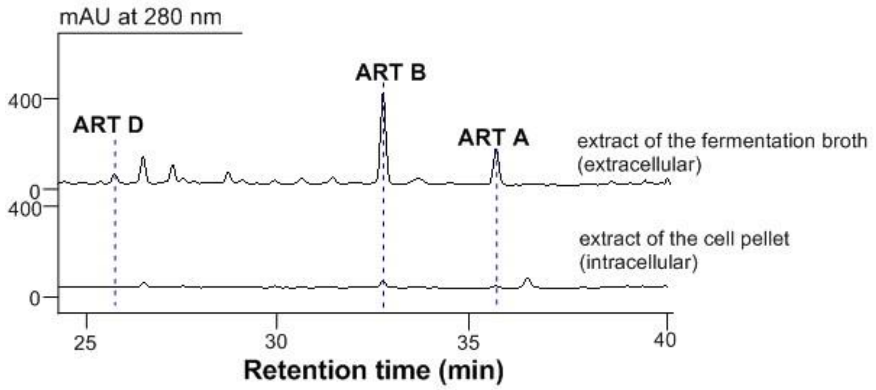

Supplementary Fig. 19. HPLC analysis of the distribution of ARTs in *B. subtilis* fmb60 culture. The results indicated that ARTs are pumped out of the cells efficiently during the fermentation.

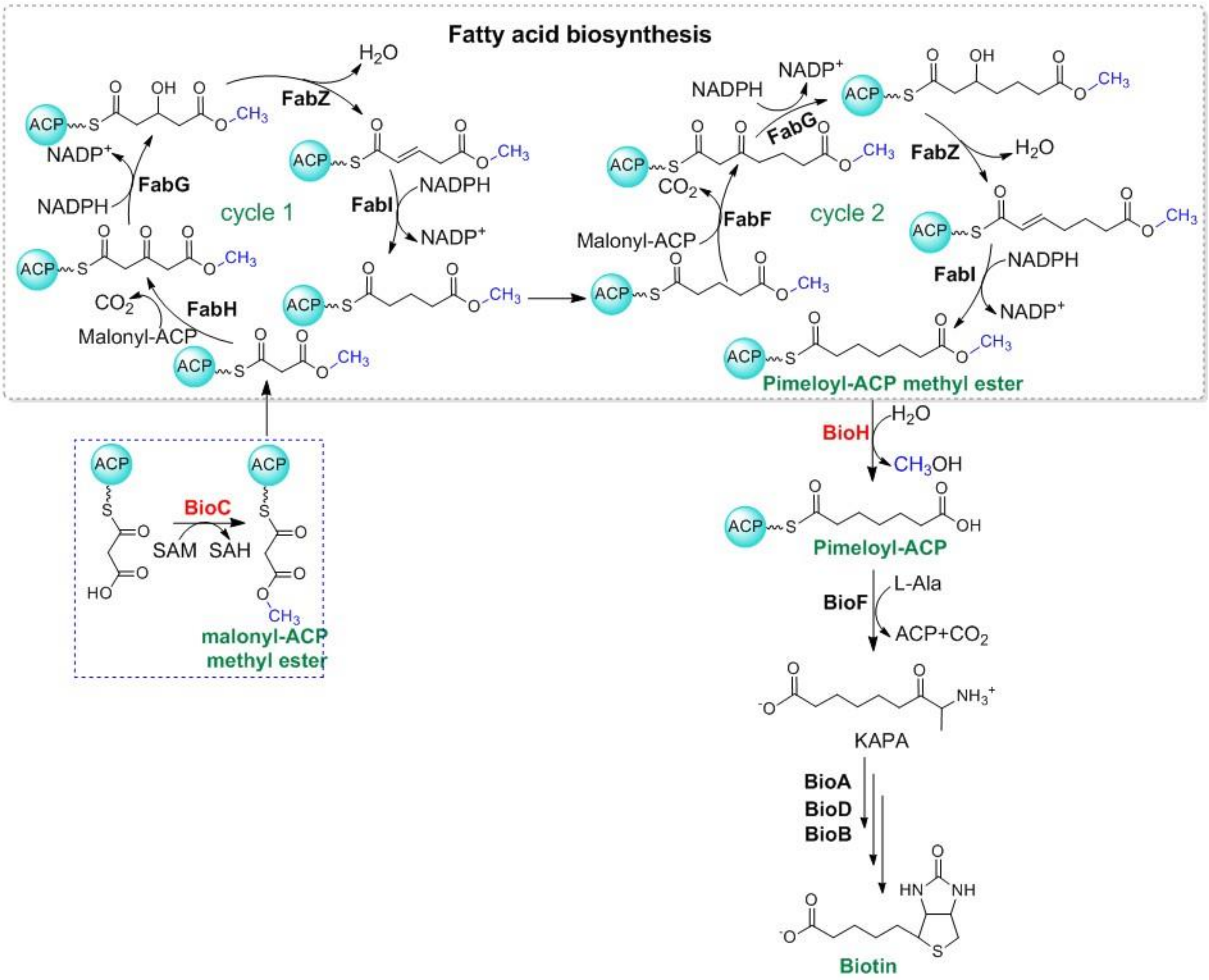

Supplementary Fig. 20. Scheme of a biotin biosynthetic pathway[5]. BioC and BioH are marked in red.

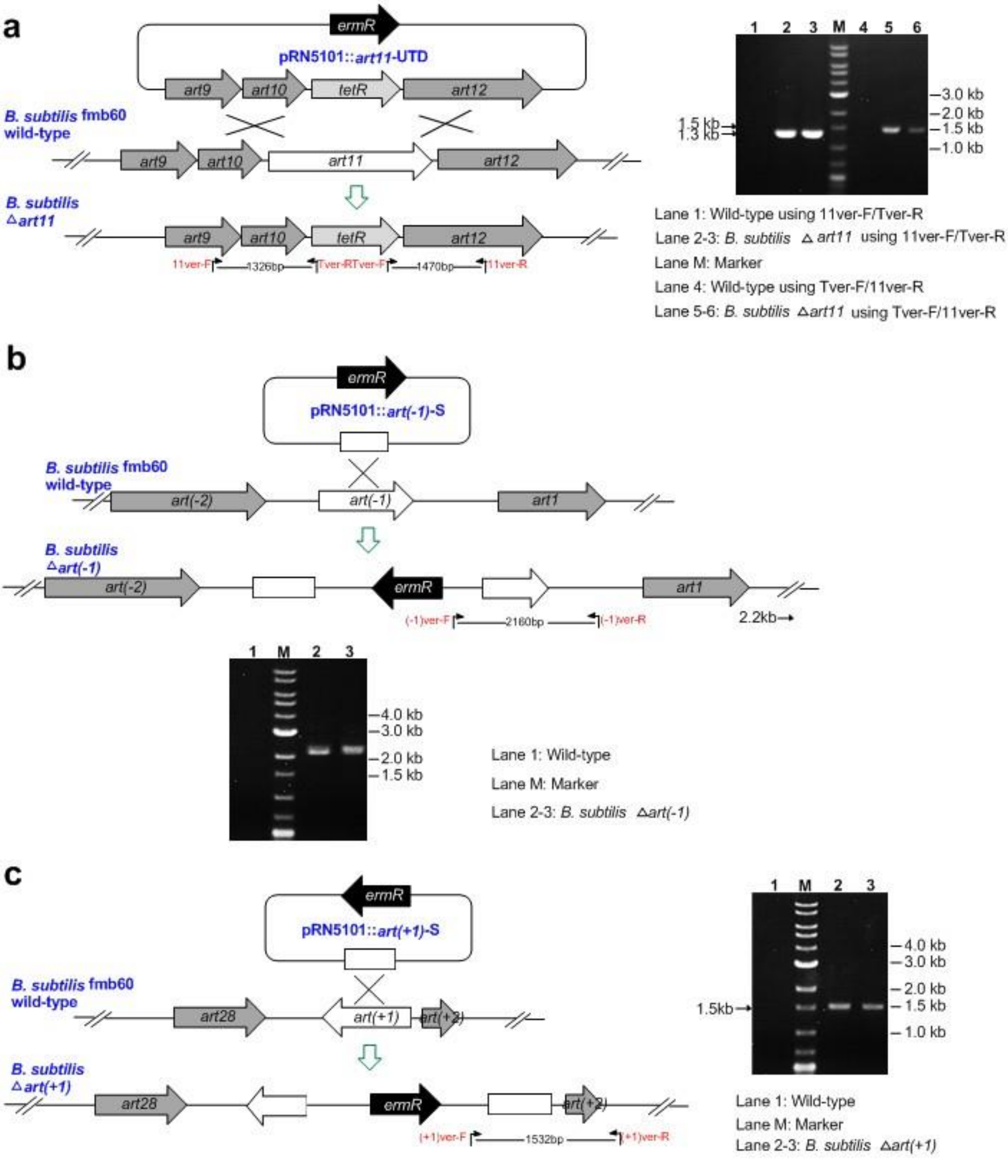

Supplementary Fig. 21. Construction of *B. subtilis* Δ*art11*, Δ*art(-1)*, and Δ*art(+1)*. (**a**) Construction of *B. subtilis* Δ*art11*. *B. subtilis* Δ*art11* was constructed by replacing gene *art11* with a tetracycline resistance gene cassette *tetR*. (**b**) Construction of *B. subtilis* Δ*art(-1)*. (**c**) Construction of *B. subtilis* Δ*art(+1)*. *B. subtilis* Δ*art(-1)* and Δ*art(+1)* were constructed using a single-crossover strategy. The primers used for genotype verification of the mutants were indicated with red and the sizes of the PCR products were also shown.

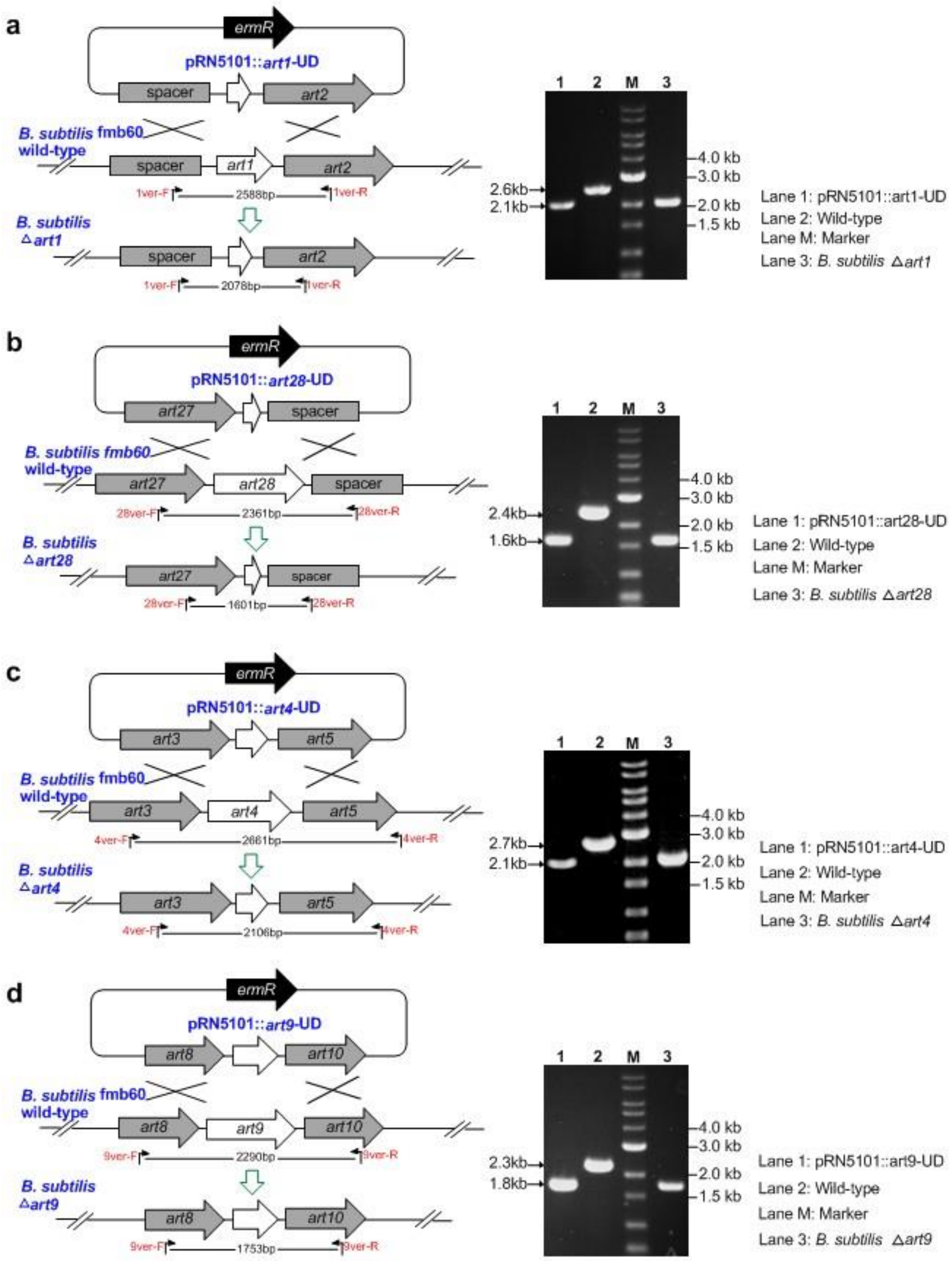

Supplementary Fig. 22. Construction of *B. subtilis* Δ*art1*, Δ*art28*, Δ*art4*, and Δ*art9*. Construction of *B. subtilis* Δ*art1* (**a**), *B. subtilis* Δ*art28* (**b**), *B. subtilis* Δ*art4* (**c**), and *B. subtilis* Δ*art9* (**d**) were performed using a gene in-frame deletion strategy. The primers used for genotype verification of the mutants were indicated with red and the sizes of the PCR products were also shown.

Supplementary references

1. Yang, J. et al. Genomics-inspired discovery of three antibacterial active metabolites, aurantinins B, C, and D from compost-associated *Bacillus subtilis* fmb60. *J. Agric. Food Chem.* **64,** 8811-8820 (2016).
2. Zhang, L. et al. CerR, a single-domain regulatory protein of the LuxR family, promotes cerecidin production and immunity in *Bacillus cereus*. *Appl. Environ. Microbiol*. **84**, e02245-17 (2018).
3. Fang, J. et al. Cloning and characterization of the tetrocarcin A gene cluster from *Micromonospora chalcea* NRRL 11289 reveals a highly conserved strategy for tetronate biosynthesis in spirotetronate antibiotics. *J. Bacteriol.* **190,** 6014-6025 (2008).
4. Lohmana, J. R. et al. Structural and evolutionary relationships of "AT-less" type I polyketide synthase ketosynthases. *Proc. Natl. Acad. Sci. U S A* **112,** 12693-12698 (2015).
5. Lin, S., Hanson, R. E. & Cronan, J. E. Biotin synthesis begins by hijacking the fatty acid synthetic pathway. *Nat. Chem. Biol*. **6,** 682-688 (2010).
