# Supplementary material for: Initiating polyketide biosynthesis by on-line methyl esterification
Source: Nat Commun. 2021 Jul 23;12:4499. doi: 10.1038/s41467-021-24846-7 (PMC8302727; doi:10.1038/s41467-021-24846-7)
Supplement: Supplementary file 3 — reporting summary [file 41467_2021_24846_MOESM3_ESM.pdf]

nature research

Corresponding author(s): Yihua Chen

Last updated by author(s): Jul 5, 2021

Reporting Summary

Nature Research wishes to improve the reproducibility of the work that we publish. This form provides structure for consistency and transparency in reporting. For further information on Nature Research policies, see our Editorial Policies and the Editorial Policy Checklist.

Statistics

For all statistical analyses, confirm that the following items are present in the figure legend, table legend, main text, or Methods section.

n/a	Confirmed	
☐	☒	The exact sample size (n) for each experimental group/condition, given as a discrete number and unit of measurement
☐	☒	A statement on whether measurements were taken from distinct samples or whether the same sample was measured repeatedly
☐	☒	The statistical test(s) used AND whether they are one- or two-sided<br>*Only common tests should be described solely by name; describe more complex techniques in the Methods section.*
☒	☐	A description of all covariates tested
☒	☐	A description of any assumptions or corrections, such as tests of normality and adjustment for multiple comparisons
☐	☒	A full description of the statistical parameters including central tendency (e.g. means) or other basic estimates (e.g. regression coefficient) AND variation (e.g. standard deviation) or associated estimates of uncertainty (e.g. confidence intervals)
☐	☒	For null hypothesis testing, the test statistic (e.g. F, t, r) with confidence intervals, effect sizes, degrees of freedom and P value noted<br>*Give P values as exact values whenever suitable.*
☒	☐	For Bayesian analysis, information on the choice of priors and Markov chain Monte Carlo settings
☒	☐	For hierarchical and complex designs, identification of the appropriate level for tests and full reporting of outcomes
☒	☐	Estimates of effect sizes (e.g. Cohen's d, Pearson's r), indicating how they were calculated

Our web collection on statistics for biologists contains articles on many of the points above.

Software and code

Policy information about availability of computer code

Data collection	All HPLC data were collected using Shimadzu HPLC system installed with LCsolution 1.25. All LC-MS data were collected using Agilent 1260/6460 Triple-Quadrupole LC/MS system installed with Qualitative Analysis B.03.01. All NMR data were collected using Bruker-500 NMR spectrometer installed with Bruker topspin 3.2. Fluorescent intensity was measured using Biotek Synergy H4 microplate reader.
Data analysis	The softwares used for data analysis are listed as follow: CLUSTALW2, antiSMASH 5.0, TMpred online tool (https://embnet.vital-it.ch/software/TMPRED_form.html), MEGA 6.0, MestReNova.9.0.1, Qualitative Analylsis B.07.00, and TransATor online tool (http://transator.ethz.ch/).

For manuscripts utilizing custom algorithms or software that are central to the research but not yet described in published literature, software must be made available to editors and reviewers. We strongly encourage code deposition in a community repository (e.g. GitHub). See the Nature Research guidelines for submitting code & software for further information.

Data

Policy information about availability of data

All manuscripts must include a data availability statement. This statement should provide the following information, where applicable:

- Accession codes, unique identifiers, or web links for publicly available datasets
- A list of figures that have associated raw data
- A description of any restrictions on data availability

The genome information of B. subtilis fmb60 is publicly available through NCBI under accession number NZ_LYMC01000002 [https://www.ncbi.nlm.nih.gov/nuccore/LYMC00000000.1/]. The antiSMASH database is publicly available [https://antismash-db.secondarymetabolites.org/]. The authors declare that all data supporting the findings of this study are available within the article and its Supplementary Information file. The source data underlying Supplementary Figures 9 and

15 are provided as a Source Data file.

Field-specific reporting

Please select the one below that is the best fit for your research. If you are not sure, read the appropriate sections before making your selection.

- [x] Life sciences
- [ ] Behavioural & social sciences
- [ ] Ecological, evolutionary & environmental sciences

For a reference copy of the document with all sections, see nature.com/documents/nr-reporting-summary-flat.pdf

Life sciences study design

All studies must disclose on these points even when the disclosure is negative.

Sample size	Sample size was determined based on standards for fermentation analysis and enzymatic assay studies, attempting to have a minimum of n=3 independent experiments with sufficient reproducibility. The results obtained show that chosen sample size was appropriate because sufficient reproducibility and clear distinctions can be seen. The fermentation of wild-type and mutant strains were carried out at least three times. All in vitro assays were performed at least three independent times.
Data exclusions	No data exclusion was performed.
Replication	The reproducibility was verified by at least three independent experiments with similar results. All attempts at replication were successful.
Randomization	Randomization is not relevant to this study because this study does not involve group allocation.
Blinding	All information about the outputs of enzymatic assays and structure identification was kept blind for the technicians who performed the LC-MS and NMR. No group allocation was involved in this study.

Reporting for specific materials, systems and methods

We require information from authors about some types of materials, experimental systems and methods used in many studies. Here, indicate whether each material, system or method listed is relevant to your study. If you are not sure if a list item applies to your research, read the appropriate section before selecting a response.

Materials & experimental systems

n/a	Involved in the study
x	Antibodies
x	Eukaryotic cell lines
x	Palaeontology and archaeology
x	Animals and other organisms
x	Human research participants
x	Clinical data
x	Dual use research of concern

Methods

n/a	Involved in the study
x	ChIP-seq
x	Flow cytometry
x	MRI-based neuroimaging
